# A novel *Actinidia* cytorhabdovirus characterized using genomic and viral protein interaction features

**DOI:** 10.1111/mpp.13110

**Published:** 2021-07-20

**Authors:** Yanxiang Wang, Guoping Wang, Jianyu Bai, Yongle Zhang, Ying Wang, Shaohua Wen, Liu Li, Zuokun Yang, Ni Hong

**Affiliations:** ^1^ Key Laboratory of Plant Pathology of Hubei Province College of Plant Science and Technology Huazhong Agricultural University Wuhan China; ^2^ Key Laboratory of Horticultural Crop (Fruit Trees) Biology and Germplasm Creation of the Ministry of Agriculture Huazhong Agricultural University Wuhan China; ^3^ Laboratory of Fruit Trees Disease Institute of Economic Forestry Xinjiang Academy of Forestry Sciences Urumqi China

**Keywords:** *Actinidia* spp., cytorhabdovirus, protein–protein interaction, subcellular location

## Abstract

A novel cytorhabdovirus, tentatively named Actinidia virus D (AcVD), was identified from kiwifruit (*Actinidia chinensis*) in China using high‐throughput sequencing technology. The genome of AcVD consists of 13,589 nucleotides and is organized into seven open reading frames (ORFs) in its antisense strand, coding for proteins in the order N‐P‐P3‐M‐G‐P6‐L. The ORFs were flanked by a 3′ leader sequence and a 5′ trailer sequence and are separated by conserved intergenic junctions. The genome sequence of AcVD was 44.6%–51.5% identical to those of reported cytorhabdoviruses. The proteins encoded by AcVD shared the highest sequence identities, ranging from 27.3% (P6) to 44.5% (L), with the respective proteins encoded by reported cytorhabdoviruses. Phylogenetic analysis revealed that AcVD clustered together with the cytorhabdovirus Wuhan insect virus 4. The subcellular locations of the viral proteins N, P, P3, M, G, and P6 in epidermal cells of *Nicotiana benthamiana* leaves were determined. The M protein of AcVD uniquely formed filament structures and was associated with microtubules. Bimolecular fluorescence complementation assays showed that three proteins, N, P, and M, self‐interact, protein N plays a role in the formation of cytoplasm viroplasm, and protein M recruits N, P, P3, and G to microtubules. In addition, numerous paired proteins interact in the nucleus. This study presents the first evidence of a cytorhabdovirus infecting kiwifruit plants and full location and interaction maps to gain insight into viral protein functions.

## Graphical abstract

The complete genome of a novel cytorhabdovirus, Actinidia virus D, was sequenced. We report for the first time that the rhabdovirus M protein shows unique microtubule localization and recruits N, P, P3, and G to microtubules.

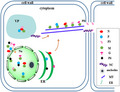

## 1 INTRODUCTION

Kiwifruit (*Actinidia* spp.) is an important horticultural crop. It is native to southwestern China (Huang & Ferguson, 2001). China is the largest kiwifruit producer, accounting for more than half (56%) of the global production (FAOstat, 2016). To date, at least 19 viruses have been reported to infect kiwifruit trees worldwide (Blouin et al., 2013; Veerakone et al., 2018; Wang et al., 2016, 2020; Zhao et al., 2019, 2020; Zheng et al., 2017).

The family *Rhabdoviridae* (order Mononegavirales) consists of numerous viruses infecting a broad range of hosts, including vertebrates, invertebrates, and plants. Viruses in the family *Rhabdoviridae* are characterized by bullet‐shaped (infecting vertebrates and invertebrates) or bacilliform (infecting plants) virions and contain a linear, negative‐sense single‐stranded RNA genome of 10.8–16.1 kb (Kuzmin et al., 2009; Walker et al., 2018). The family *Rhabdoviridae* includes three subfamilies and seven genera (Kuhn et al., 2020). Members of the subfamily *Betarhabdovirinae* are classified into six genera (*Alphanucleorhabdovirus*, *Betanucleorhabdovirus*, *Cytorhabdovirus*, *Dichorhavirus*, *Gammanucleorhabdovirus*, and *Varicosavirus*) based on their replication sites (nucleus or cytoplasm), genome structures (monopartite or bipartite), and vector species (Freitas‐Astúa et al., 2019; Jackson et al., 2005; Walker et al., 2018). Cytorhabdoviruses encode at least six canonical proteins, including nucleocapsid protein (N), phosphoprotein (P), movement protein (P3), matrix protein (M), glycoprotein (G), and RNA‐dependent RNA polymerase (L), in the order 3′‐N‐P‐P3‐M‐G‐L‐5′ (Dietzgen et al., 2020; Walker et al., 2018). In the enveloped particles of viruses in the family *Rhabdoviridae*, protein N encapsulates the viral genomic RNA to generate N–RNA complexes, and each complex together with proteins P and L forms a ribonucleoprotein (RNP) complex, which is the minimal infectious unit. Protein M plays roles in the condensation of RNP complexes into a skeleton‐like structure (RNP‐M core) during virion assembly, and protein G forms transmembrane spikes (Assenberg et al., 2010; Jackson et al., 2005). In addition, some cytorhabdoviruses encode one or more accessory proteins between the coding regions of P and M and/or G and L (Walker et al., 2011). Understanding the interaction and location maps of viral proteins is necessary for revealing the functions of viral genes and the components involved in the assembly of virions. To date, there are only two cytorhabdoviruses, lettuce necrotic yellows virus (LNYV) and alfalfa dwarf virus (ADV), for which the subcellular localizations and interactions between proteins have been described (Bejerman et al., 2015; Martin et al., 2012).

High‐throughput sequencing (HTS) technologies are powerful tools for the identification of known and novel viruses (Bejerman et al., 2020; Navarro et al., 2018; Rott et al., 2017). In recent years, at least 17 cytorhabdoviruses have been discovered by using HTS (Bejerman et al., 2020). Most cytorhabdoviruses were identified from poaceous crops and herbaceous plants. Cytorhabdoviruses infecting woody plants include persimmon virus A from persimmon (*Diospyros kaki*) in Japan (Ito et al., 2013), papaya virus E from papaya (*Carica papaya*) in Ecuador (Medina‐Salguero et al., 2019), and paper mulberry mosaic‐associated virus from paper mulberry (*Broussonetia papyrifera*) in China (Qiu et al., 2020). Papaya virus E has also been reported from citrus (*Citrus* spp.) and passion fruit (*Passiflora edulis*) in China (Zhang et al., 2021). Cytorhabdoviruses can be vectored by aphids (Aphididae), planthoppers (Cicadellidae), leafhoppers (Delphacidae), and whiteflies (Aleyrodidae) in a circulative and propagative manner (Dietzgen et al., 2020; Ng & Perry, 2004; Pinheiro‐Lima et al., 2020; Whitfield et al., 2018; Yang et al., 2016).

Previously, in order to obtain a complete overview of the kiwifruit virome, we constructed six RNA sequencing (RNA‐seq) libraries from 69 kiwifruit samples (Wen et al., 2020). During a re‐examination for viruses in these HTS data, we identified one contig that partially matched the genome sequences of some cytorhabdoviruses. Then, the genome of the virus was determined by Sanger sequencing. The virus has a genomic structure that is typical of viruses in the family *Rhabdoviridae* and is phylogenetically related to cytorhabdoviruses. Furthermore, the intracellular localization and interaction maps of the viral proteins were determined.

## 2 RESULTS

### 2.1 Identification and genomic organization of Actinidia virus D

During the BLASTX searches using the assembled contig sequences from six RNA‐seq libraries of kiwifruit samples against the NCBI data set, we identified a large contig (ID: contig100_2) of 13,579 nucleotides (nt) from library JS with significant amino acid (aa) sequence identity of 21.5%–44.8% to proteins encoded by cytorhabdoviruses (Table 1). The identified contig was further aligned along the best matched genomic RNA of Wuhan insect virus 4 (WhIV4, accession no. KM817650) to generate a preliminary genomic organization of a potential novel cytorhabdovirus, provisionally named Actinidia virus D (AcVD) following the previously reported kiwifruit virus names. Total RNA was extracted from each of the four samples (JS27, JS29, JS30, and JS45) included in the RNA‐seq library (JS). Reverse transcription (RT)‐PCR using the virus‐specific primers NF/NR (Table S1) and sequencing of amplified products confirmed the presence of AcVD in two samples (JS27 and JS29). The 1,363‐bp amplicons generated from the two samples shared 99.4% sequence identity with each other and the corresponding sequence of contig100_2. Then, the sample JS27 was used for further viral genome characterization.

**TABLE 1:** BLASTX and bioinformatics analyses of proteins encoded by Actinidia virus D (AcVD)

ORF	Position (nt)	Size (nt)	Protein	Size (aa)	MW (kDa)	pI^a^	BLASTX hit^b^	Identity (%)	TM^c^	NLS (Score)	NES^e^
1	160–1,596	1,437	N	468	53.8	5.40	WhIV4	42.4	ND	Bipartite (2.8)	ND
2	1,732–2,646	915	P	304	34.1	4.12	WhIV4	39.1	ND	ND	IQLK_240–243_, I_246_
3	2,768–3,862	1,095	P3	364	40.2	9.16	WhIV4	43.9	ND	Monopartite (3.0)	ND
4	3,981–4,553	573	M	190	21.0	9.40	CcYV‐1	21.5	ND	Bipartite (3.2)	ND
5	4,793–6,451	1,659	G	552	62.5	6.61	WhIV4	33.5	aa 5–27, 507–529	ND	L_323_
6	6,560–6,742	183	P6	60	6.9	9.07	—	—	aa 13–33	ND	IKFIAM_26–31_, L_33_
7	6,888–13,235	6,318	L	2,115	243.2	7.18	WhIV4	44.8	ND	Monopartite (7.0)	L_964_, LSL_968–970_, L_972_, I_2014_

^a^ pI, isoelectric point.

^b^ WhIV4, Wuhan insect virus 4; CcYV‐1, cabbage cytorhabdovirus 1; —, no hit.

^c^ TM, transmembrane; ND, not detected.

^d^ NLS, nuclear localization signal.

^e^ NES, nuclear export signal.

**FIGURE 1:**
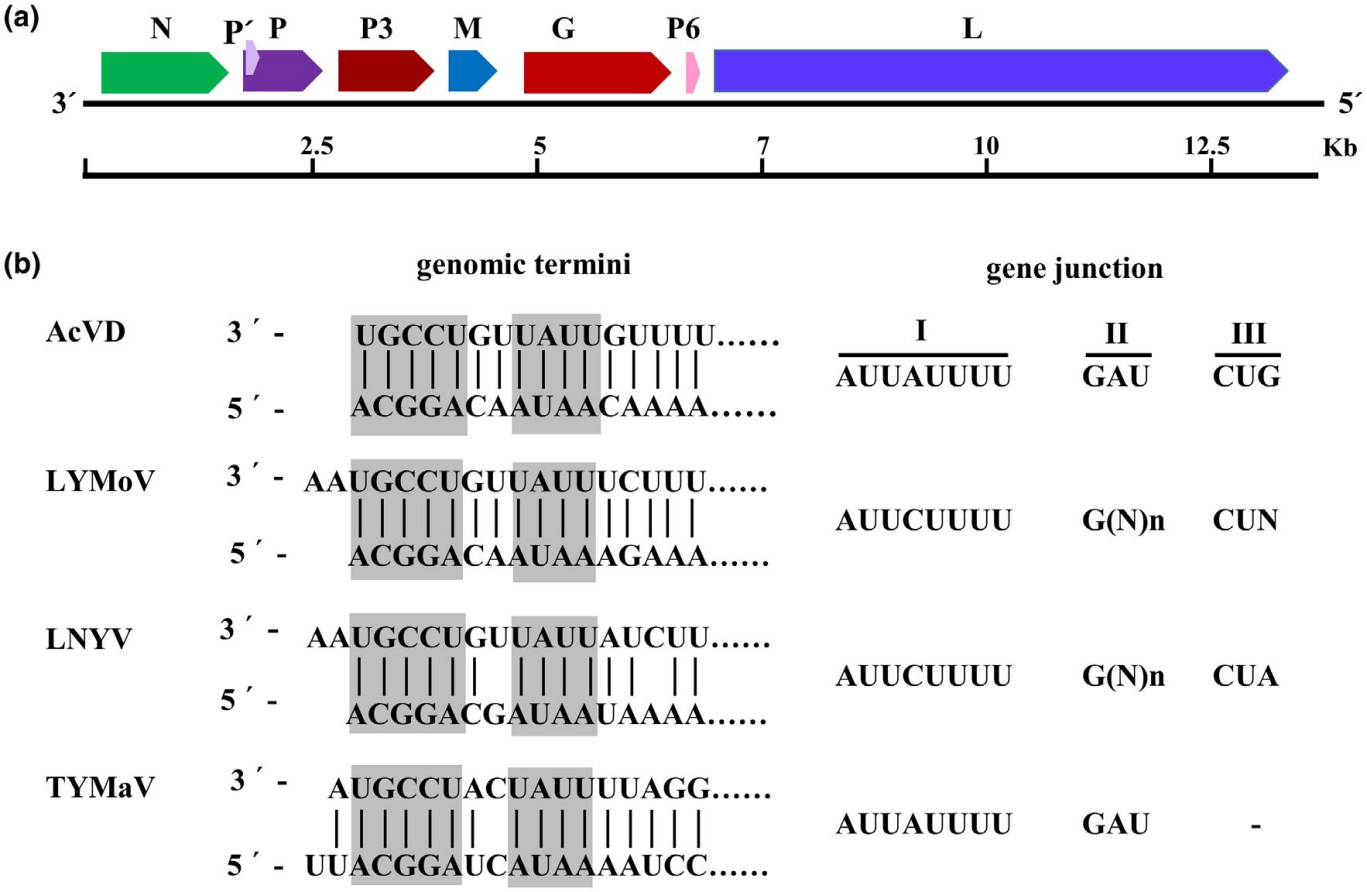
Genome organization (a), complementary terminal nucleotides, and conserved gene junctions of Actinidia virus D (AcVD) and selected cytorhabdoviruses (b). In (a), open reading frames (ORFs) are represented by coloured and arrowed rectangles, and the encoded protein is labelled above each ORF. An approximate size scale (kb) is shown below the RNA genome. In (b), vertical lines indicate complementary nucleotides between leader and trailer sequences. Abbreviations of referred viruses and their GenBank accession numbers are as follows: LNYV, lettuce necrotic yellows virus (AJ867584); LYMoV, lettuce yellow mottle virus (EF687738); TYMaV, tomato yellow mottle‐associated virus (KY075646)

The full‐length genome of AcVD consists of 13,589 nt (GenBank accession no. MW550041), and seven open reading frames (ORFs) were identified in its anti‐sense strand (Figure 1a). The proteins encoded by ORF1–5 and ORF7 matched well with the nucleocapsid protein (N), phosphoprotein (P), movement protein (P3), matrix protein (M), glycoprotein (G), and RNA‐dependent RNA polymerase (L) of reported cytorhabdoviruses, and protein P6 (encoded by ORF6) had no hit with proteins deposited in the NCBI database (Table 1). The 3′ leader and the 5′ trailer of the AcVD genome were 159 nt and 354 nt, respectively. The 5′ and 3′ termini of the AcVD genomic RNA were well complementary to each other for their first 16 nt, similar to other cytorhabdoviruses (Figure 1b). Additionally, the first 30 nt of the 3′ leader sequence had a high nt U content of 50%, similar to those of bean‐associated cytorhabdovirus (47%), strawberry crinkle cytorhabdovirus (SCV) (47%), raspberry vein chlorosis virus (RVCV) (60%), LNYV (60%), and lettuce yellow mottle virus (LYMoV) (63%).

Similar to all plant viruses in the family *Rhabdoviridae*, the AcVD ORFs are separated by a highly conserved gene junction with the consensus sequence 3′‐AUUAUUUUGAUCUG‐5′, which is composed of a 3′ polyadenylation signal of the preceding gene (element I), an intergenic spacer (element II), and a transcription initiation sequence of the following gene (element III) (Figure 1b), which is one of the typical features of the genomes of viruses in the family *Rhabdoviridae* (Jackson et al., 2005). The sequences of elements I and II of the AcVD gene junction are similar to those of tomato yellow mottle‐associated virus (TYMaV), while element III is almost identical to those of LNYV and LYMoV (Figure 1b).

ORF1 is 1,437 nt in length and encodes a putative N protein of 468 aa with a predicted molecular weight (MW) of 53.8 kDa and an isoelectric point (pI) of 5.40 (Table 1). Pairwise comparisons revealed sequence identities ranging from 39.2% to 52.9% (nt) and from 17.4% to 40.1% (aa) between ORF1 and orthologues of reported cytorhabdoviruses (Table 2). Protein N contains a predicted bipartite nuclear localization signal (NLS) (score = 2.8, aa position 21–52) at the N‐terminal region (Table 1).

**TABLE 2:** The nucleotide and amino acid sequence identities (%) between Actinidia virus D (AcVD) and reported cytorhabdoviruses

Virus^a^	Genome	N		P		P3		M		G		P6/P9		L	
	nt%	nt%	aa%	nt%	aa%	nt%	aa%	nt%	aa%	nt%	aa%	nt%	aa%	nt%	aa%
WhIV4	**51.5**	**52.9**	**40.1**	**48.7**	**28.6**	49.7	**36.4**	44.3	19.2	44.6	**29.1**	37.5	18.8	**54.6**	**44.5**
TpVB	47.8	47.6	35.9	42.3	20.7	48.2	30.1	44.6	18.9	41.8	25.7	‐	‐	50.8	40.2
LNYV	48.4	46.3	31.6	46.9	23.5	46.0	33.7	40.9	20.0	43.3	28.7	‐	‐	50.9	40.6
SCV	47.3	48.0	30.0	44.8	23.8	41.7	19.5	**45.0**	16.6	24.5	26.0	42.1	11.5	50.6	37.2
CCyV‐1	47.6	47.8	32.3	43.5	22.8	**50.0**	32.9	**45.0**	**20.9**	42.8	27.2	‐	‐	49.9	38.5
LYMoV	47.7	46.9	32.9	35.7	19.9	46.6	31.3	**45.0**	16.5	43.2	26.0	‐	‐	49.9	40.3
TYMaV	47.0	44.4	26.4	43.1	19.3	36.6	12.5	40.5	14.9	43.3	27.2	26.9	8.3	49.8	36.3
WhIV6	47.1	49.0	29.7	44.9	22.0	40.1	11.7	44.0	17.6	43.1	28.3	36.2	‐	50.1	37.7
TpVA	47.1	46.0	27.8	32.6	21.2	38.7	11.8	40.0	14.3	24.5	27.4	**48.9**	17.1	50.0	38.1
ADV	47.0	46.2	30.3	37.8	18.8	31.7	12.2	41.2	18.5	43.1	26.5	35.5	11.2	50.0	37.3
RVCV	47.0	48.2	29.6	45.3	19.7	38.2	13.6	39.3	18.3	43.2	26.1	45.1	7.1	50.1	37.2
PeVA	48.8	46.9	28.8	45.2	19.2	33.8	12.6	38.8	14.8	44.1	27.4	‐	‐	52.2	39.5
WhIV5	47.3	45.9	26.0	44.5	20.5	38.2	4.5	37.6	19.7	44.0	29.4	‐	‐	50.5	38.8
BYSMV	45.6	39.2	21.2	39.1	14.6	29.4	12.3	44.3	11.6	23.7	20.7	48.3	23.4	46.9	29.8
MYSV	45.3	41.6	21.6	45.3	16.7	42.7	10.4	42.1	16.5	42.5	23.1	47.6	25.0	46.9	29.3
NCMV	44.6	40.2	20.0	45.7	18.9	32.8	9.1	39.3	16.0	43.3	20.1	28.9	22.6	46.8	28.2
MaCyV	44.2	42.4	19.5	41.7	19.7	33.6	8.6	43.8	16.0	**45.4**	18.2	‐	‐	47.3	28.3
RSMV	45.1	44.9	18.8	40.3	11.6	31.6	6.0	40.9	1.0	43.4	22.6	48.8	13.4	46.8	28.8
CBDaV	44.7	44.0	18.7	44.8	18.8	33.1	5.3	40.8	20.6	24.2	19.8	43.4	**27.3**	46.4	28.7
YmCaV	44.6	41.0	19.6	43.7	10.0	32.3	7.9	42.8	5.1	41.1	18.5	‐	‐	45.9	26.3
PpVE	44.9	43.3	17.4	36.5	13.1	34.9	10.2	40.6	11.8	43.7	19.0	‐	‐	48.7	27.0
CuCV‐1	45.0	42.2	17.5	36.3	16.8	36.3	11.3	43.8	4.3	41.2	18.8	‐	‐	45.9	27.1
PuMaV	44.6	41.4	20.9	40.5	12.0	32.8	6.4	43.3	5.6	44.1	19.0	‐	‐	47.4	29.5
StrV‐1	45.5	46.0	26.4	42.3	19.3	37.7	10.8	40.5	18.2	23.0	27.9	‐	‐	48.7	35.7
TrARV‐1	47.3	50.0	28.9	44.8	19.7	40.2	12.0	43.9	21.2	41.6	28.6	‐	‐	49.7	38.9

^a^ Wuhan insect virus 4 (WhIV4) [KM817650]; Trifolium pratense virus B (TpVB) [MH982249]; lettuce necrotic yellows virus (LNYV) [AJ867584]; strawberry crinkle cytorhabdovirus (SCV) [MH129615]; cabbage cytorhabdovirus 1 (CCyV‐1) [KY810772]; lettuce yellow mottle virus (LYMoV) [EF687738]; tomato yellow mottle‐associated virus (TYMaV) [NC_034240.1]; Wuhan insect virus 6 (WhIV6) [KM817652]; Trifolium pratense virus A (TpVA) [MH982250]; alfalfa dwarf virus (ADV) [KP205452]; raspberry vein chlorosis virus (RVCV) [MK257717]; persimmon virus A (PeVA) [NC_018381]; Wuhan insect virus 5 (WhIV5) [KM817651]; barley yellow striate mosaic virus (BYSMV) [KM213865]; maize yellow striate virus (MYSV) [KY884303]; northern cereal mosaic virus (NCMV) [MH282832]; maize‐associated cytorhabdovirus (MaCyV) [KY965147]; rice stripe mosaic virus (RSMV) [MH720469]; Colocasia bobone disease‐associated virus (CBDaV) [KT381973]; yerba mate chlorosis‐associated virus (YmCaV) [KY366322]; papaya virus E (PpVE) [MK202584], Trichosanthes‐associated rhabdovirus 1 (TrARV1) [BK011194], paper mulberry mosaic‐associated virus (PMuMaV) [MN872813], cucurbit cytorhabdovirus 1 (CuCV‐1) [MT381995], strawberry cytorhabdovirus 1 (StrV‐1) [MK21127].

ORF2 is 915 nt in length and encodes a putative P protein of 304 aa with a predicted MW of 34.1 kDa (pI = 4.12) (Table 1). Protein P of AcVD shares identities ranging from 32.6% to 48.7% (nt) and from 11.6% to 28.6% (aa) with orthologues of reported cytorhabdoviruses (Table 2). Two putative nuclear export signals (NESs) at aa positions 240–243 and 246 were found in protein P of AcVD (Table 1). A small overlapping ORF corresponding to P′ was predicted for AcVD. The P′ ORF encodes a small 6.3‐kDa protein (51 aa) that contains a predicted transmembrane domain (aa positions 13–35).

ORF3 is 1,095 nt in length and encodes a putative polypeptide (P3) of 364 aa with a predicted MW of 40.2 kDa (pI = 9.16) (Table 1). Sequence identities between ORF3 and corresponding proteins of reported cytorhabdoviruses range from 31.7% to 50.0% (nt) and from 4.5% to 36.4% (aa) (Table 2). One NLS was predicted at the C‐terminal region (IDKKRREYSK_353–362_) of AcVD protein P3 (Table 1). CCD and SMART analyses revealed that AcVD P3 belongs to the 30K‐like viral movement protein (MP) family (pfam01107; interval: 21–199; E‐value: 8.4e−22). Sequence alignments of P3 proteins from AcVD, ADV, and LNYV and the 30K superfamily MPs of two tobamoviruses (tobacco mosaic virus [TMV] and turnip vein‐clearing virus) using the PROMALS3D program showed that AcVD P3 harbours a D_116_ residue of the 30K protein‐specific LxD/N_50−70_G motif and has a secondary structure that is similar to that of the 30K superfamily plant virus MPs (Figure S1).

ORF4 is 573 nt in length and encodes a putative M protein of 190 aa with a predicted MW of 21.0 kDa (pI = 9.40) (Table 1). The M protein of AcVD shares the highest sequence identities (45.0% [nt] and 20.9% [aa]) with cabbage cytorhabdovirus 1 M protein (Table 2). Additionally, a putative bipartite NLS (score = 3.2) at the C‐terminal region (aa positions 152–183) was found in protein M of AcVD (Table 1).

ORF5 is 1,659 nt in length and encodes a putative G protein of 552 aa with a predicted MW of 62.5 kDa (pI = 6.61) (Table 1). It shared sequence identities ranging from 23.0% to 45.4% (nt) and from 18.2% to 29.1% (aa) with the G proteins of other cytorhabdoviruses (Table 2). The sequence of protein G contains a 23‐aa signal peptide at the N‐terminal region, an NES (L_323_), and four predictive glycosylation sites (NTT_205–207_, NPS_343–345_, NYT_427–429_, and NLS_465–467_). In addition, two transmembrane domains located at the N‐terminal (aa positions 5–27) and C‐terminal regions (aa positions 507–529) were predicted (Table 1).

ORF6 is 183 nt in length and encodes a small basic (pI = 9.07), hydrophobic protein (P6) of 60 aa with a predicted MW of 6.9 kDa (Table 1). The ORF6 of AcVD shares the highest nt sequence identity (48.9%) with ORF6 of Trifolium pratense virus A and the highest aa sequence identity (27.3%) with P6 of Colocasia bobone disease‐associated virus (Table 2). Like P6/P9 proteins encoded by other viruses in the family *Rhabdoviridae*, protein P6 of AcVD has high isoleucine and/or leucine content (20%). Eight potential phosphorylation sites (S_8_, S_10_, S_12_, Y_16_, T_36_, Y_39_, S_42_, T_46_, and T_50_) and two NESs (IKFIAM_26–31_ and L_33_) were predicted in protein P6 of AcVD. Additionally, membrane topology predictions indicated that P6 contains a short N‐terminal luminal domain (aa positions 1–12), a central transmembrane domain (aa positions 13–33), and a longer C‐terminal cytoplasmic domain (aa positions 34–60) that is rich in basic residues.

ORF7 is 6,318 nt in length and encodes a putative L protein of 2,115 aa with a predicted MW of 243.2 kDa (pI = 7.18) (Table 1). The L protein of AcVD shows sequence identities ranging from 45.9% to 54.6% at the nt level and from 26.3% to 44.5% at the aa level with those of other cytorhabdoviruses (Table 2). Three conserved domains, including Mononeg_RNA_pol (aa positions 149–1,083; E‐value: 2.26e−131), Mononeg_mRNAcap (aa positions 1,098–1,335; E‐value: 4.26e−50), and G‐7‐MTase (aa positions 1,194–1,802; E‐value: 3.63e−48) were recognized in the L protein sequence of AcVD by SMART and CCD analyses. Additionally, alignment of L proteins of AcVD and other plant viruses in the family *Rhabdoviridae* revealed that the AcVD L protein harbours six motifs that are conserved in RNA‐dependent RNA polymerases of negative‐sense single‐stranded RNA viruses (Figure S2) and a GHP (Gly‐His‐Pro) motif. One NLS (GLPKRKRVCL_55–64_) and six NESs (L_964_, LSL_968–970_, L_972_, and I_2014_) were predicted in the L protein (score 7.0) (Table 1).

### 2.2 Phylogenetic analysis

**FIGURE 2:**
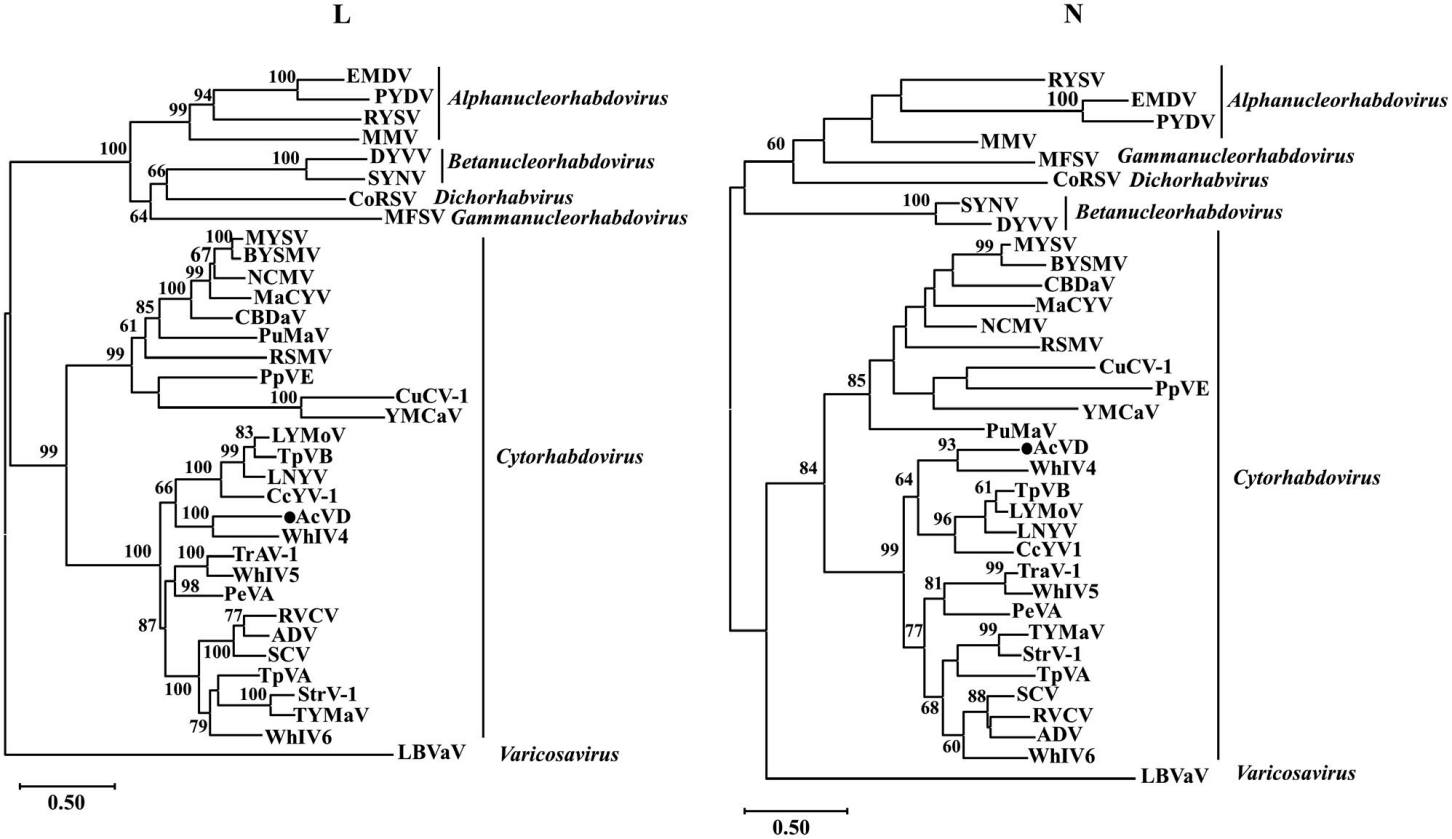
Unrooted maximum‐likelihood phylogenetic trees generated based on the deduced amino acid sequences of proteins L and N of Actinidia virus D (AcVD) and other plant viruses in the family *Rhabdoviridae*. The bar represents the number of amino acid replacements per site. AcVD is highlighted by a black dot. GenBank accession numbers and abbreviations of viruses used for phylogenetic analyses are shown in Table S2

Phylogenetic analyses for AcVD proteins N and L and homologous proteins of 25 reported cytorhabdoviruses, 4 alphanucleorhabdoviruses, 2 betanucleorhabdoviruses, 1 gammanucleorhabdovirus, 1 dichorhavirus, and 1 varicosavirus revealed that AcVD clustered together with WhIV4 within the cytorhabdovirus group with a high bootstrap value (>99) (Figure 2).

### 2.3 Subcellular localization of AcVD proteins in *Nicotiana benthamiana* leaf cells

**FIGURE 3:**
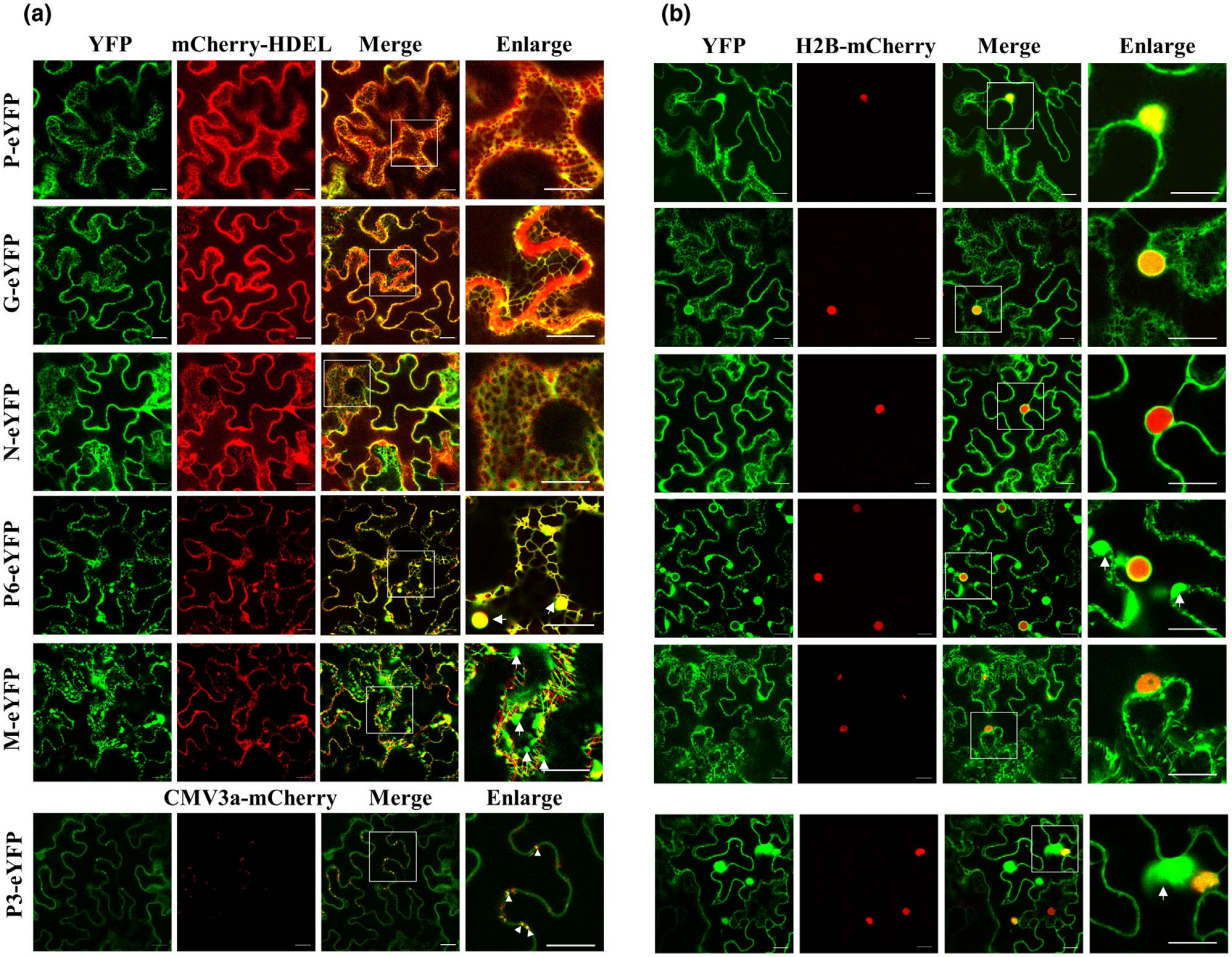
Subcellular localization assays of Actinidia virus D (AcVD) proteins P, G, N, P6 M, and P3 in epidermal cells of *Nicotiana benthamiana* leaves. The six viral proteins were expressed as fusions to the N‐terminus of eYFP. The fusion proteins mCherry‐HDEL, H2B‐mCherry, and CMV3a‐mCherry, were used as endoplasmic reticulum (ER), nucleus, and plasmodesma (PD) markers, respectively. The aggregated bodies formed by proteins P3, M, and P6 are denoted by arrows. Punctate dots indicating colocalization of AcVD‐P3 with CMV3a‐mCherry at PDs are highlighted by arrow heads. The enlarged section of each image is highlighted with a box. Images were acquired at 2 days after agroinfiltration under a confocal microscope using a 63×/1.20 water objective. Scale bar = 20 μm

When the viral proteins N, P, P3, M, G, and P6 were individually fused to the N‐terminus of an enhanced yellow fluorescent protein (eYFP), at 2 days post‐agroinfiltration (dpi), the fluorescence signals of P‐eYFP, G‐eYFP, N‐eYFP, and P6‐eYFP were observed in the periphery of agroinfiltrated *Nicotiana benthamiana* leaf cells and colocalized well with the fused endoplasmic reticulum (ER) marker protein mCherry‐HDEL (Figure 3a). In addition, P‐eYFP and G‐eYFP also localized in the nucleus, and weak signals of N‐eYFP and P6‐eYFP were observed in the nucleus, as validated using the nuclear marker protein H2B‐mCherry, and P6‐eYFP also formed aggregated bodies with variable sizes in the cell periphery (Figure 3b). Interestingly, M‐eYFP formed numerous fluorescent bodies in the cytoplasm and clearly filamentous structures, which were not colocalized with the ER network (Figure 3a). The M‐eYFP protein was also nuclear‐localized, as validated using the nuclear marker protein H2B‐mCherry (Figure 3b). P3‐eYFP colocated at the plasmodesma (PD) with the marker protein CMV3a‐mCherry as punctate spots along cell walls, and also accumulated in the nucleus and formed aggregates in the cytoplasm (Figure 3b). When these proteins were fused to the C‐terminus of eYFP, the same location features were observed for proteins N, P, P6, M, and P3, but only a weak fluorescent signal in the cell periphery and nucleus was observed for eYFP‐G (Figure S3). All these location features were different from the fluorescent signal of free eYFP in cytoplasm and nucleus (Figure S4).

### 2.4 Protein M of AcVD is associated with microtubules

**FIGURE 4:**
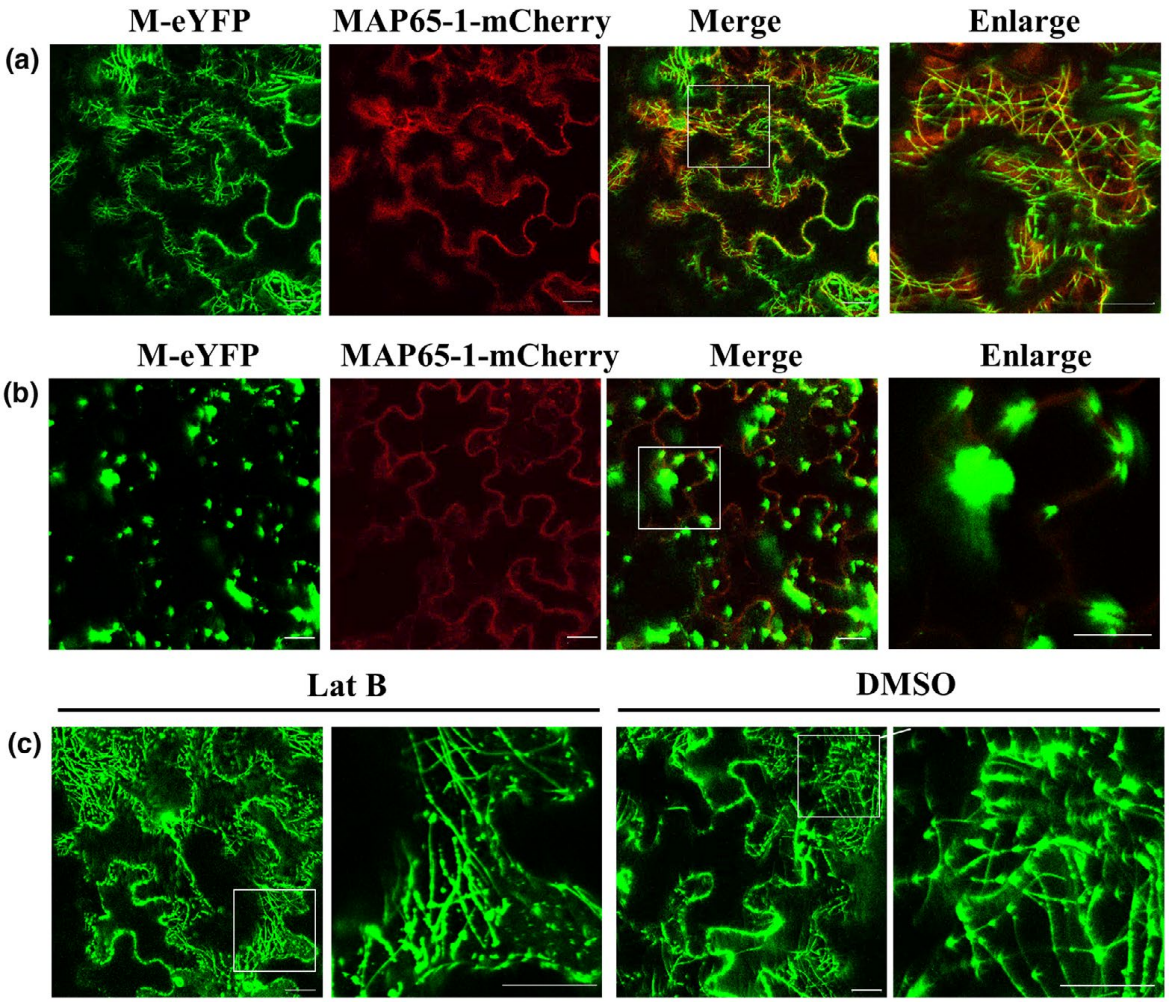
The microtubule localization of Actinidia virus D (AcVD) protein M in epidermal cells of *Nicotiana benthamiana* leaves. (a) YFP‐tagged AcVD‐M colocalized with mCherry‐tagged MAP65‐1 at microtubules. (b) The microtubule‐depolymerizing agent oryzalin (20 μM) disrupted the microtubule localization of AcVD‐M. (c) The microtubule localization of AcVD‐M was not affected by the microfilament‐depolymerizing agent latrunculin B (Lat B; 5 μM) or dimethyl sulphoxide (DMSO; solvent). The enlarged section of each image is highlighted with a box. The fusion protein MAP65‐1‐mCherry was used as a microtubule marker. Images were acquired at 2 days after agroinfiltration under a confocal microscope using a 63×/1.20 water objective. Scale bar = 20 μm

Given that the filamentous structures of M‐eYFP in the cytoplasm were similar to microtubules, we tested the potential association of M protein with microtubules by coexpression of M‐eYFP with the microtubule marker MAP65‐1‐mCherry. At 2 dpi, MAP65‐1‐mCherry labelled microtubules, which colocalized with the filamentous structures of M protein (Figure 4a), suggesting that M‐eYFP was associated with microtubules of *N. benthamiana* cells. To further test whether microtubules are necessary for the formation of the M‐eYFP filamentous structures, we treated *N. benthamiana* leaves with 20 μM oryzalin at 2 days after coinfiltration of M‐eYFP and MAP65‐1‐mCherry. At 3 hr after oryzalin treatment, the filamentous structures of M‐eYFP were completely disrupted and appeared as many large bodies in the cytoplasm (Figure 4b). In contrast, treatment with latrunculin B (Lat B) or dimethyl sulphoxide (DMSO) did not affect the location pattern of M‐eYFP (Figure 4c). These results indicated that the M protein of AcVD was associated with microtubules, but not with microfilaments.

### 2.5 Homologous and heterologous interactions of AcVD proteins

**FIGURE 5:**
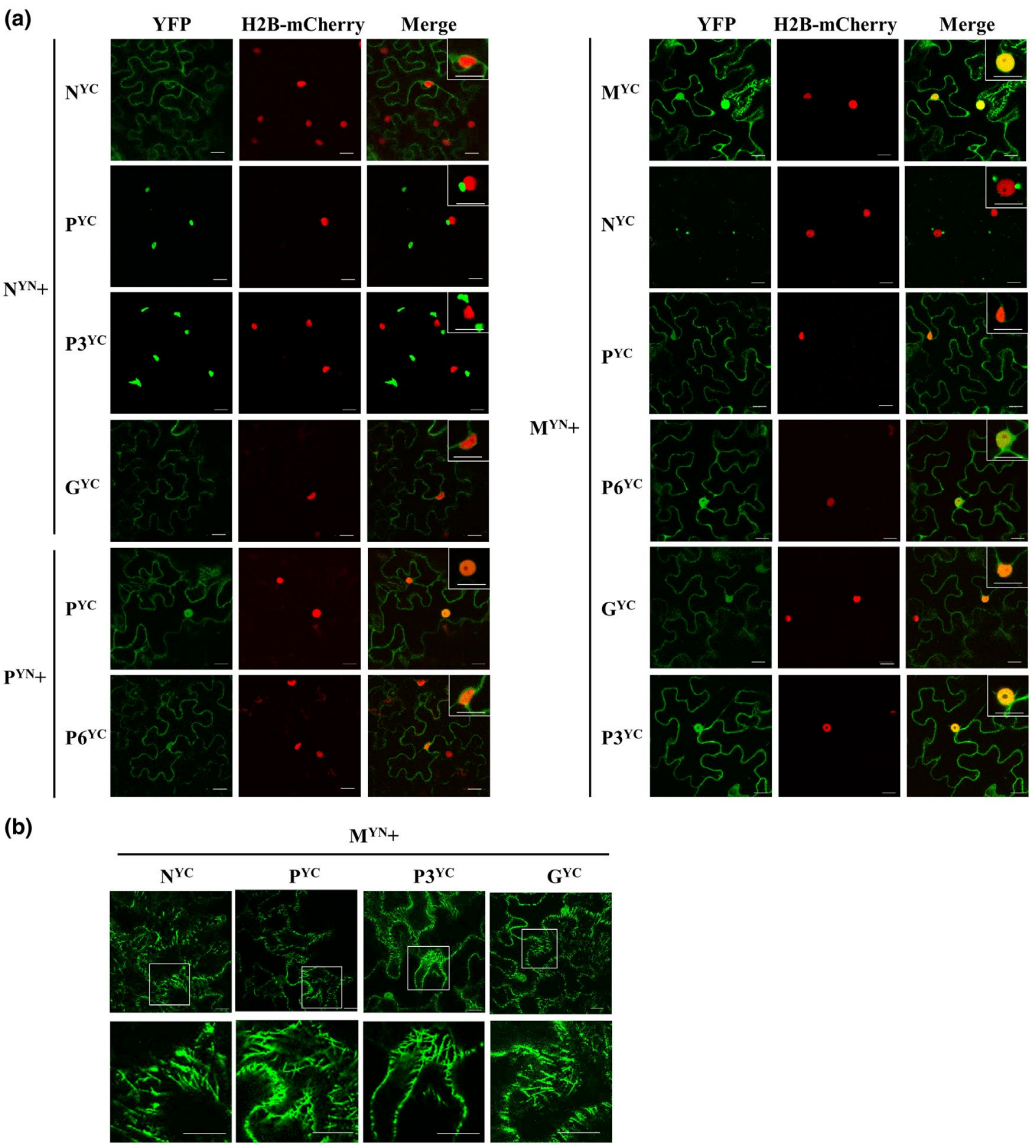
Bimolecular fluorescence complementation (BiFC) assays of the protein–protein interactions of Actinidia virus D (AcVD) in *Nicotiana benthamiana* epidermal cells. (a) Homologous and heterologous interactions of paired AcVD proteins. The H2B‐mCherry fusion protein was used as a nuclear marker and the boxes at the upper right corner of merged images show magnifications of the inclusion bodies adjacent to the nucleus or the BiFC signals in the nucleus. (b) The BiFC signals of M protein interacting with N, P, P3, or G resemble microtubule structures. Images were acquired at 2 days after agroinfiltration under a confocal microscope using a 63×/1.20 water objective. Scale bar = 20 μm

Bimolecular fluorescence complementation (BiFC) assays were performed to determine the interactions of paired AcVD proteins in *N. benthamiana* leaves. Homologous interactions were identified for proteins N, P, and M with interaction signals occurring in the cell periphery for protein N, in the cell periphery and the nucleus for protein P, and in the cell periphery, the nucleus, and filamentous structures for protein M (Figure 5). The interaction location of each protein was similar to its subcellular location. Protein N could interact with four proteins (P, P3, G, and M). The interaction of N^YN^ and G^YC^ occurred in the cell periphery, while the interaction combinations N^YN^–P^YC^, N^YN^–P3^YC^, and N^YC^–M^YN^ formed several bodies with variable sizes in the cytoplasm and/or near the nucleus of agroinfiltrated *N. benthamiana* cells, but did not colocalize with the nucleus (Figure 5a). A weak interaction signal of N^YC^–M^YN^ was also detected in the nucleus of some cells (Figure S5a). These results indicated that protein N could translocate proteins P, P3, and M by forming interaction inclusions in the cytoplasm. Besides its interaction with protein N, protein M also could interact with proteins P, P6, G, and P3 in the nucleus and the cell periphery. When green fluorescence signals were captured in lower leaf cell layers, the fluorescence signals of interaction pairs M^YN^–N^YC^, M^YN^–P^YC^, M^YN^–P3^YC^, and M^YN^–G^YC^ also accumulated as microtubule‐like structures (Figure 5b), which are highly similar to the location pattern of protein M. The interaction between P and P6 also occurred in the cell periphery and the nucleus (Figure 5a). Among these interaction combinations, only the interactions of N–P, M–P, and M–P6 were detected in both orientations in BiFC assays (Figures 5 and S5b). None of the tested AcVD proteins produced an interaction signal with glutathione S‐transferase in either orientation (Figure S5b).

**FIGURE 6:**
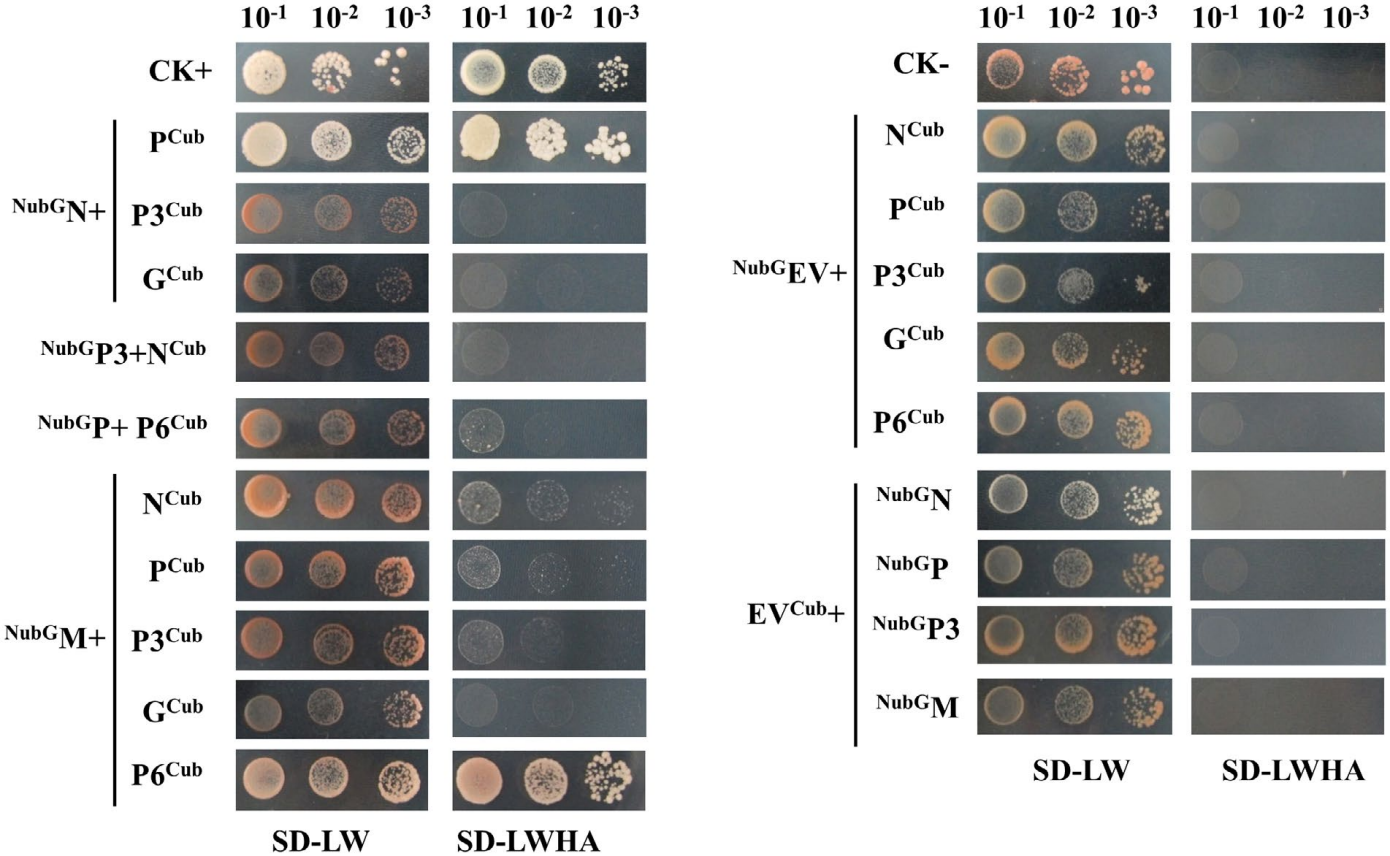
Split‐ubiquitin yeast two‐hybrid assays for Actinidia virus D (AcVD) proteins. Proteins N, P, P3, G, and P6 were individually fused to the C‐terminal half of ubiquitin (Cub) of vector pDHB1, and proteins N, P, P3, and M were individually fused to the N‐terminal half of ubiquitin (NubG) of vector pPR3‐N. Yeast cotransformed with pDHB1‐largeT/pDSL‐P53 was used as a positive control, and yeast cotransformed with pDHB1‐largeT/pPR3‐N was used as a negative control. Yeast growth on synthetic dropout medium lacking leucine and tryptophan (SD−LW) was used to confirm the presence of both plasmids. Medium lacking leucine, tryptophan, histidine, and adenine (SD−LWHA) was used to screen for positive interactions. A series of dilutions (10^−1^, 10^−2^, and 10^−3^) is shown

**FIGURE 7:**
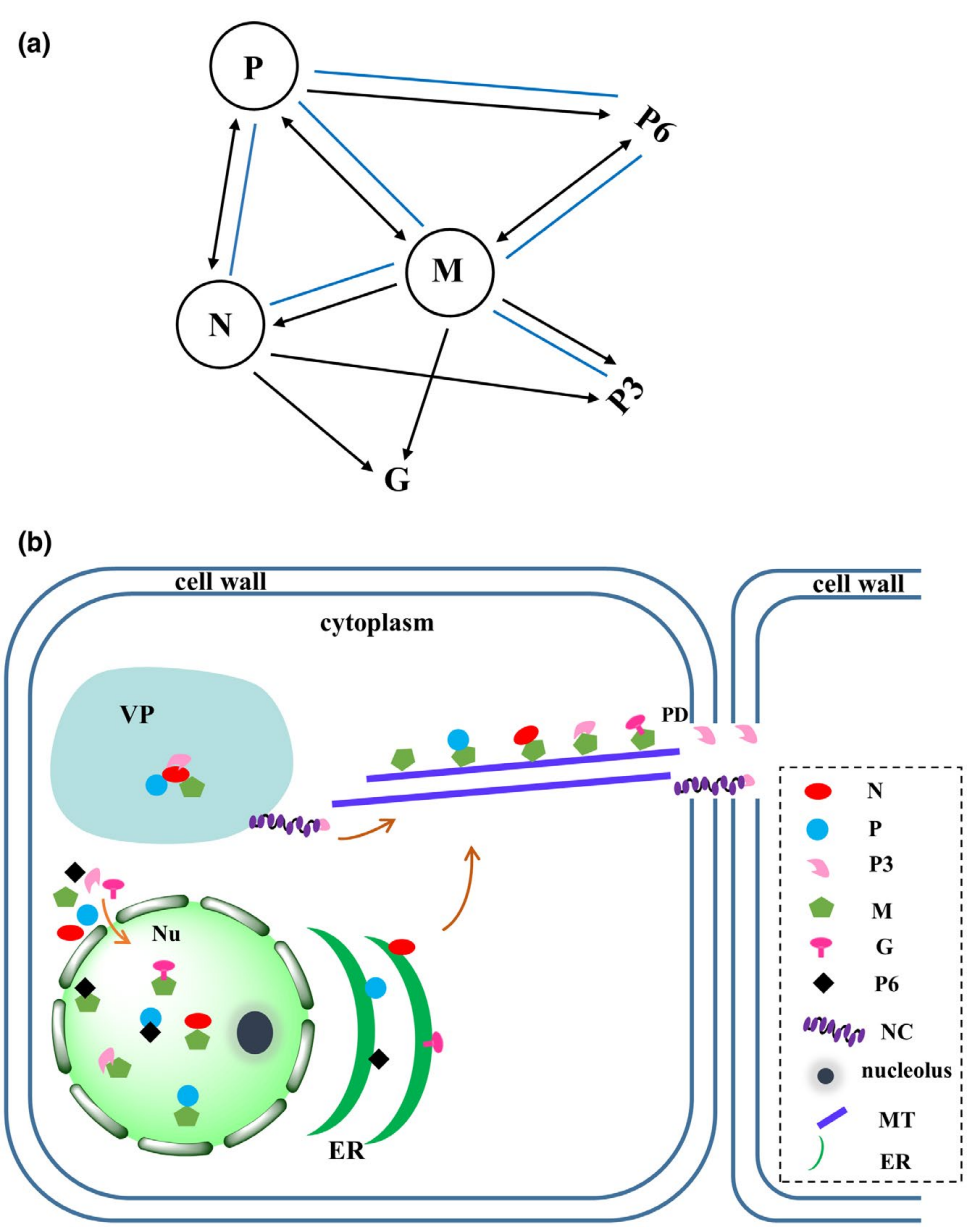
Integrated protein interaction map and model for viroplasm (VP) formation and translocation of Actinidia virus D (AcVD) proteins. (a) Integrated protein interaction map for AcVD based on our bimolecular fluorescence complementation (BiFC) and yeast two‐hybrid assays. Self‐interaction is indicated by a circle. The lines with single and double arrowheads indicate interactions in one and two orientations in our BiFC assay, respectively. The blue solid lines indicate heterologous interactions as confirmed by our yeast two‐hybrid assay. (b) The VP contains complexes of N–P, N–P3, and N–M, which formed in the cytoplasm in our BiFC assays. The viral RNA genome is encapsidated by protein N and then associates with proteins P and L to form minimal infectious unit nucleocapsids (NCs). Protein M recruits the NC core proteins N and P and the movement protein P3 to the microtubules (MTs) to guide the complex intracellular and intercellular trafficking through plasmodesmata (PDs). The interactions of M–N, M–P, M–P3, M–G, M–P6, and P–P6 occurring in the nucleus (Nu) might play regulatory roles. The endoplasmic reticulum (ER) locations of four proteins (N, P, G, and P6) might be involved in their intracellular transportation

The heterologous interactions of AcVD proteins were further confirmed by split‐ubiquitin‐based membrane yeast two‐hybrid (MYTH) assay. The results showed that yeast cells transformed with paired vectors ^NubG^N/P^Cub^, ^NubG^P/P6^Cub^, ^NubG^M/N^Cub^, ^NubG^M/P^Cub^, ^NubG^M/P3^Cub^, and ^NubG^M/P6^Cub^ were able to grow on synthetic dropout medium lacking adenine, tryptophan, histidine, and alanine (SD−LWHA), while yeast cells transformed with paired vectors ^NubG^N/P3^Cub^, ^NubG^P3/N^Cub^, ^NubG^N/G^Cub^, and ^NubG^M/G^Cub^ and all transformants with a viral proteins paired with the empty vector (EV) did not grow (Figure 6). Moreover, yeast cells transformed with ^NubG^P/P6^Cub^, ^NubG^M/N^Cub^, ^NubG^M/P^Cub^, and ^NubG^M/P3^Cub^ grew slowly, indicating that weak interactions occurred between these paired proteins. Thus, six out of nine interaction pairs identified in BiFC assays were confirmed by MYTH assays (Figure 7a).

### 2.6 The infection status of AcVD in kiwifruit

RT‐PCR assays using the two sets of primers 1F/1R and 7F/7R revealed that four *A. chinensis* samples (IDs: JS17, JS22, JS24, and JS33) from the same location in Hubei Province were positive for AcVD, and 167 other kiwifruit leaf samples were negative for the virus. Sequence analysis showed that the 847‐bp fragment of the viral ORF1 amplified using primer set 1F/1R from these samples shared 99.3%–99.8% nt sequence identity and 98.6%–100% aa sequence identity, and the 544‐bp fragment of the viral ORF7 amplified using primer set 7F/7R showed 98.9%–100% sequence identities at both nt and aa levels.

## 3 DISCUSSION

In recent years, four new viruses belonging to families *Fimoviridae* (Zheng et al., 2017), *Closteroviridae* (Veerakone et al., 2018), and *Betaflexiviridae* (Blouin et al., 2018; Zhao et al., 2020) have been identified from kiwifruit by HTS technologies. In the present study, we characterized a novel virus, AcVD, from kiwifruit at the molecular level using HTS combined with conventional Sanger sequencing. AcVD has a genomic organization with seven ORFs in its antisense strand, coding for proteins in the order N‐P‐P3‐M‐G‐P6‐L, which is similar to the genomic structure of cytorhabdoviruses ADV, TYMaV, RVCV, SCV, and rice stripe mosaic virus (Bejerman et al., 2015; Fránová et al., 2019; Jones et al., 2019; Koloniuk et al., 2018; Xu et al., 2017; Yang et al., 2016), and a tentative cytorhabdovirus Trifolium pratense virus A (Fránová et al., 2019). AcVD shared the highest genomic sequence identity (51.5%) with WhIV4, and its seven ORFs showed aa identities of <50% with counterparts of cytorhabdoviruses available in the GenBank database. In the phylogenetic trees inferred from aa sequences of proteins L and N, the virus clustered together with WhIV4 within the cytorhabdovirus group. According to the newly revised criteria for species demarcation in the genus *Cytorhabdovirus*, which states that genomic sequence identity is less than 75% and aa sequence identity of all cognate ORFs is less than 80% for different species (Freitas‐Astúa et al., 2019) 2019, AcVD should be considered as a new species in the genus *Cytorhabdovirus*. To the best of our knowledge, this is the first report of a cytorhabdovirus infecting kiwifruit plants.

The low occurrence frequency and limited occurrence location of AcVD indicates a recent infection of the virus in kiwifruit. Because the six kiwifruit plants (including two samples subjected to RNA‐seq analysis) positive for AcVD are also infected by some other viruses (Wen et al., 2020), the coinfection of AcVD with other viruses makes it not possible to evaluate the association of the virus with kiwifruit disease. The cytorhabdovirus group mainly consists of viruses from plants except for three Wuhan insect viruses (WhIV4–6), which are associated with aphids and might be acquired by insects from the host plants (Li et al., 2015; Xu et al., 2017). The close phylogenetic relationship between AcVD and WhIV4 indicates that AcVD might be transmitted by aphids, because among plant viruses in the family *Rhabdoviridae* there is a strong correlation between phylogenetic relationships and their insect vectors (Dietzgen et al., 2020; Whitfield et al., 2018).

Cytorhabdoviruses replicate in the cytoplasm of infected host plant cells (Jackson et al., 2005). Cytorhabdovirus replication, including uncoating, viral protein synthesis, and viroplasm formation, is associated with ER membranes (Dietzgen et al., 2017; Jackson et al., 2005). The ER localization of four AcVD proteins (N, P, G, and P6) indicated their likely functional involvement in viral replication. We also found that AcVD proteins P, P3, M, and G accumulated in the nucleus, although no NLS was predicted in proteins P and G. Likewise, no NLS was detected in protein P of the cytorhabdovirus ADV and the alphanucleorhabdovirus potato yellow dwarf virus, but the cognate proteins from the two viruses were located in the nucleus (Bandyopadhyay et al., 2010; Bejerman et al., 2015). The location of AcVD protein P in both the cell periphery and the nucleus is similar to that of two beta‐ and a gammanucleorhabdovirus, namely datura yellow vein virus (DYVV), sonchus yellow net virus (SYNV), and maize fine streak virus (Dietzgen et al., 2015; Goodin et al., 2001; Tsai et al., 2005), and different from the exclusive cytoplasmic localization of LNYV protein P and/or the nuclear localization of ADV protein P (Bejerman et al., 2015; Martin et al., 2012). Previous studies showed that neither ADV protein P nor SYNV protein P could enter the nucleus by interacting with importin‐α, and P proteins of ADV and SYNV have RNA silencing suppressor activity (Bejerman et al., 2016; Deng et al., 2007; Jackson et al., 2005; Krichevsky et al., 2006; Martin et al., 2009). Elucidation of the nuclear import pathway and the specific function of AcVD protein P requires further study. The P3 protein of AcVD has an aspartic acid (D) residue in the conserved LxD/N_50–70_G motif in the β2 strand of the 30K MP family of plant viruses and displays PD subcellular localization, supporting that it is probably the MP of AcVD. Like the P3 protein of AcVD, the MPs of five plant viruses in the family *Rhabdoviridae*, including LNYV, ADV, DYVV, maize mosaic virus (MMV), and coffee ringspot virus, are also localized in the nucleus of *N. benthamiana* cells (Bejerman et al., 2015; Dietzgen et al., 2015; Martin et al., 2012; Martin & Whitfield, 2018; Ramalho et al., 2014). The AcVD M protein is uniquely associated with microtubules, in contrast to the reported nuclear and/or ER localization of M proteins of some plant and animal viruses in the family *Rhabdoviridae* (Bandyopadhyay et al., 2010; Bejerman et al., 2015; Dietzgen et al., 2015; Martin et al., 2012; Martin & Whitfield, 2018; Ramalho et al., 2014; Tsai et al., 2005). The AcVD P6 protein is located on the ER network and forms inclusion‐like bodies along the cell membrane or in the cytoplasm, which was different from the reported ER or nuclear localization of P6/P9 of the cytorhabdoviruses ADV and barley yellow striate mosaic virus (Bejerman et al., 2015; Yan et al., 2015). We also noticed that the ER localization observed for the G‐eYFP fusion protein was not clear for eYFP‐G, possibly because the different fusion orientations could affect the accession or interaction of the viral protein with plant targets, as reported for ADV, MMV, and SYNV (Bejerman et al., 2015; Goodin et al., 2007; Martin & Whitfield, 2018).

Previous studies showed that the N–P and M–M interactions are common to some analysed viruses in the family *Rhabdoviridae* (Bandyopadhyay et al., 2010; Ge et al., 2010; Graham et al., 2008; Martin et al., 2012). The N–P and M–M interactions are also conserved for AcVD. Our study showed a complex interaction map of the AcVD‐encoded proteins in BiFC assays. The interaction combinations N–P3, N–G, and M–G identified in BiFC assays were not detected in the MYTH assays (Figure 7a). During in vivo BiFC assays, plant proteins might serve as bridges in the interaction between two exogenous proteins (Martin & Whitfield, 2018; Min et al., 2010; Paape et al., 2006). In addition, different vectors used in the yeast two‐hybrid assay could also lead to different results because the fusion directions may affect protein folding or function (Zhou et al., 2019a). Previous studies revealed that some cellular factors are involved in cell‐to‐cell movement of SYNV, LNYV, and ADV by mediating viral protein interactions (Mann et al., 2016; Min et al., 2010). Interestingly, the interaction of N with P, P3, or M resulted in the formation of numerous aggregates in the cytoplasm, and some interaction complexes were found to be closely adjacent to the nucleus. Similar perinuclear bodies have been identified as viral replication complexes (VRCs) (Barton et al., 2017). The N–P interaction of LNYV also results in the formation of aggregations in the cytoplasm (Martin et al., 2012). Considering the potential function of P3 in viral movement, the N–P3 interaction bodies formed in the BiFC assay might be involved in viral movement. Cytorhabdoviruses and nucleorhabdoviruses are distinguished based on their viral replication and particle assembly sites in the cytoplasm or the nucleus, respectively (Jackson et al., 2005). The N protein functions in the encapsidation of viral genomic RNA and is a component of the viroplasm (Jackson et al., 2005). Probably, the N protein of AcVD recruits the two structural proteins P and M and the movement protein P3 to VRC‐like or inclusion‐like bodies in the cytoplasm. These findings support the notion that the viral N protein interacts with two nucleocapsid core proteins (P and M) and the movement protein P3 to form MP‐nucleocapsid complexes (Figure 7b). The cell biological evidence for the interaction of protein N with these proteins together with the genome sequence supports the assignment of AcVD as a cytorhabdovirus. The interaction between N and MP was also described for an alphanucleorhabdovirus (rice yellow stunt virus [RYSV]) (Huang et al., 2005), a betanucleorhabdovirus (SYNV) (Huang et al., 2005; Zhou et al., 2019a), a cytorhabdovirus (TYMaV) (Zhou et al., 2019a), and some orthotospoviruses (Diwaker et al., 2015; Leastro et al., 2015; Leastro et al., 2017; Tripathi et al., 2015; Widana & Dietzgen, 2017). The MP of tomato spotted wilt virus can facilitate the movement of the RNP complex through interaction with N protein (Kormelink et al., 1994; Soellick et al., 2000). The microtubule cytoskeleton is involved in the transport of materials within cells. Many plant viruses use the plant cytoskeleton for virion and VRC movement from the replication site to PDs (Naghavi & Walsh, 2017). The association of viral MPs with microtubules is necessary for the intracellular movement of plant viruses (Boyko et al., 2000; Nicolas & Manfred, 2018; Niehl et al., 2013). The disruption of microtubules can prevent TMV movement (Boutant et al., 2009; Ferralli et al., 2006; Niehl et al., 2013). The M protein of the alphanucleorhabdovirus RYSV interacts with leafhopper tubulin to facilitate viral transport (Wang et al., 2019). The MPs of LNYV and SYNV could interact with a microtubule‐associated protein, NbVOZ1, implicating that NbVOZ1 might aid viral movement (Mann et al., 2016; Min et al., 2010). The M protein of viruses in the family *Rhabdoviridae* is thought to participate in nucleocapsid coiling and in connecting the capsid to the viral glycoprotein(s) inserted in the lipid bilayer (Assenberg et al., 2010; Jackson et al., 2005; Sun et al., 2018). The microtubule location of AcVD protein M might increase the chances for its interactions with other viral proteins during the viral life cycle. The interactions of M with N, P, P3, and G were all detected in the cell periphery, nucleus, and microtubules, which are similar to the location of M alone. Thus, our results suggest that AcVD protein M could recruit three structural proteins (N, P, and G) and the movement protein P3 to the microtubule network (Figure 7b), which might be necessary for virion assembly and intracellular trafficking of movement complexes (Assenberg et al., 2010; Fondong, 2013; Jackson et al., 2005; Zhou et al., 2019a). The nuclear location of protein M of viruses in the family *Rhabdoviridae* might play a role in blocking the export of host mRNAs in the infected cells through interactions with nuclear export proteins (Faria et al., 2005) and limiting competition of resources for the viral proteins (Carroll & Wagner, 1979; Clinton et al., 1978; De et al., 1982; Finke & Conzelmann, 2003; Von Kobbe et al., 2000; Zhou et al., 2019b). In the nucleus of plant cells, the AcVD protein M interacts with five nuclear‐localized proteins (N, P, P3, G, and P6) (Figure 7b). This localization may be linked to the recruitment of host transcription factors, as reported for SYNV (Min et al., 2010), or some other specific functions. The function of P6 proteins of viruses in the family *Rhabdoviridae* is poorly understood. RYSV P6 is thought to be associated with virions in viruliferous aphids and to function as an RNA silencing suppressor (Guo et al., 2013; Jackson et al., 2005). The P6 protein of AcVD harbours the structural characteristics of class 1a viroporins, which are commonly present in mammalian viruses in the family *Rhabdoviridae* (Walker et al., 2011), and interacts with the P and M proteins. Viroporins participate in virus particle assembly and promote viral particle release from cells (Nieva et al., 2012; Walker et al., 2015). Thus, the functions of P6–M and P6–P interactions could be linked with these functions.

In conclusion, here we present the complete genome sequence of AcVD, which has the same genomic organization as the viruses in the genus *Cytorhabdovirus* and shows low genomic sequence identities with reported cytorhabdoviruses. The virus has a low occurrence frequency in kiwifruit. A full subcellular location map and the interaction network of six AcVD proteins are reported. Our BiFC assays provide biological evidence that the viral protein N interacts with three other viral proteins (P, P3, and G) and forms viroplasms in the cytoplasm of *N. benthamiana* leaf cells. Moreover, we report for the first time that the M protein of a virus in the family *Rhabdoviridae* shows unique microtubule localization and that it could recruit four viral proteins (N, P, P3, and G) to microtubules.

## 4 EXPERIMENTAL PROCEDURES

### 4.1 Virus source

To obtain a complete overview of the kiwifruit virome in China, six libraries prepared from total RNA of leaf samples from 69 kiwifruit plants were subjected to next‐generation sequencing in our previous work (Wen et al., 2020). During a re‐examination for other potential viruses in the library data sets (SRA ID: PRJNA681158), a large contig (ID: contig100_2) sharing limited amino acid sequence identities with the proteins of reported cytorhabdoviruses was identified from the JS library. Thus, the four samples (JS27, JS29, JS30, and JS45) included in the JS library were tested for the presence of the potential cytorhabdovirus using the primer set NF/NR (Table S1), which was designed based on the sequence of contig100_2. After a preliminary identification of a candidate cytorhabdovirus (tentatively named AcVD), sample JS27, which was positive for the virus, was used for amplification of the viral genome.

### 4.2 Amplification of the viral genome

Seven primer sets, including the primer set NF/NR, were designed based on the sequences of contig100_2 and employed to amplify the remaining viral sequence (Table S1). Commercial kits for rapid amplification of cDNA ends (RACE) (Takara) were used to determine the 5′ and 3′ terminal sequences of the viral genome. The 5′RACE reaction was conducted according to the manufacturer's protocol. For 3′RACE, poly(A) tails were added to the 3′ ends of the total RNAs using the poly(A) polymerase kit (Takara), and cDNA was generated using the oligo(dT) primer provided in the 3′RACE kit.

Total RNA was extracted from leaf samples (100 mg) using a cetyltrimethylammonium bromide (CTAB) method as described previously (Li et al., 2008). The resulting RNAs were used as templates for the synthesis of first‐strand cDNA using M‐MLV reverse transcriptase (Promega) and random hexamer primers pd(N)_6_ (Takara). The solutions and conditions of PCRs were similar to those previously described (Wang et al., 2016), except that the extension time and annealing temperature varied depending on the sizes of the amplicons and primer pairs used.

PCR products were gel‐purified and ligated into the pMD18‐T vector (Takara). At least three positive clones of each amplicon were sequenced at Sangon Biological Engineering & Technology and Service Co. Ltd. (Shanghai, China). The obtained sequences were assembled into contiguous sequences by overlapping common regions (>100 bp) of the adjacent amplicons with a sequence identity of >99%.

### 4.3 Sequence analysis

The potential ORFs were predicted using the ORFfinder tool on the NCBI website (https://www.ncbi.nlm.nih.gov/orffinder/; accessed 17 July 2020) with a minimal ORF length of 75 nt. The aa sequence, MW, and pI were computed using SnapGene Viewer v. 4.1.7 (GSL Biotech) with default parameters. Pairwise sequence alignments were performed using the Needle program on the EBI website (https://www.ebi.ac.uk/Tools/psa/emboss_needle/; accessed on 20 October 2020) with default settings. Conserved domains were determined utilizing the NCBI Conserved Domain Database (https://www.ncbi.nlm.nih.gov/Structure/cdd/docs/cdd_search.html/; accessed 17 July 2020) (Marchler‐Bauer et al., 2015) and the SMART tool (http://smart.embl‐heidelberg.de/; accessed 17 July 2020) (Letunic et al., 2015) with default parameters. The potential cleavage sites were predicted using SignalP v. 4.1: (http://www.cbs.dtu.dk/services/SignalP/; accessed 17 July 2020), transmembrane topology was predicted using Phobius (https://phobius.sbc.su.se/; accessed 17 July 2020) and TMHMM Server 2.0 (http://www.cbs.dtu.dk/services/TMHMM/; accessed 17 July 2020), and glycosylation sites were predicted using NetNGlyc 1.0 (http://www.cbs.dtu.dk/services/NetNGlyc/; accessed 17 July 2020). The NLSs and NESs were predicted using the cNLS Mapper (Kosugi et al., 2009) with a cut‐off score of 2.0 and the NetNES 1.1 server (La Cour et al., 2004) with default parameters. The secondary structure prediction and alignments of the MP were carried out using PROMALS3D online (http://prodata.swmed.edu/promals3d/promals3d.php; accessed 5 November 2020) with default settings (Pei & Grishin, 2014). For phylogenetic analysis, the aa sequences were aligned using the MAFFT server (https://mafft.cbrc.jp/alignment/server/; accessed 11 May 2021). Subsequently ambiguously aligned regions were trimmed with the Gblocks 0.91b server (Talavera & Castresana, 2007) (http://www.vardb.org/vardb/analysis/gblocks.html; accessed 11 May 2021) with the stringency levels lowered for all parameters. The resulting alignments with 844 aa (accounting for 31% of the original 2,684 aa of L) and 144 aa (accounting for 18% of the original 766 aa of N) were used to generate phylogenetic trees using MEGAX (Kumar et al., 2018). ML trees were constructed based on the aa sequences of proteins L and N using the best‐fit models LG+I+G+F and LG+G, respectively, with 1,000 bootstrap replications. GenBank accession numbers of virus sequences used for the phylogenetic analyses are given in Table S2.

### 4.4 Subcellular localization and BiFC assays for viral proteins in planta

A binary vector pCNY containing an eYFP gene was used for subcellular localization experiments. Six ORFs (missing stop codons) of AcVD were fused to the N‐terminus of eYFP between the *Xba*I and *Bam*HI digestion sites and to the C‐terminus of eYFP between the *Sal*I and *Sma*I digestion sites.

For BiFC assays, the primers used for the amplification of ORF1–6 of AcVD were flanked with *Att*B recombination sites at their 5′ ends to facilitate subsequent Gateway vector construction (Table S3). Amplicons were gel‐purified and cloned into vector pDONR/ZEO (Invitrogen) using BP Clonase II Enzyme Mix (Invitrogen). Sequence‐validated entry clones (without a stop codon) of these ORFs were individually recombined into binary destination vectors pEarleygate201‐YN and pEarleygate202‐YC (Lu et al., 2010) using LR Clonase II Enzyme Mix (Invitrogen).

The plasmids of mCherry‐HDEL (Nelson et al., 2007), H2B‐mCherry (Martin et al., 2009), CMV3a‐mCherry (Ur Rehman et al., 2019), and MAP65‐1‐mCherry (Smertenko et al., 2004) were used as ER, nucleus, PD, and microtubule markers, respectively. All the constructs were transformed into *Agrobacterium tumefaciens* GV3101 (Weidi Bio) using a heat shock method. *A. tumefaciens* cells were infiltrated into *N. benthamiana* leaves as described previously (Ur Rehman et al., 2019). The infiltrated leaf sections were viewed by confocal laser scanning microscopy (CLSM; TCS‐SP8, Leica Microsystems) with an HC PL APO CS2 63×/1.20 water objective at 2 dpi.

### 4.5 Depolymerization of microtubules and microfilaments

Stock solutions of oryzalin and Lat B (Sigma‐Aldrich) in DMSO were prepared at concentrations of 100 mM and 5 mM. Then, these stock solutions were diluted to 20 µM and 5 µM for microtubule and microfilament depolymerization treatments. To evaluate the effect of oryzalin on the intracellular localization of AcVD M protein, the microtubule marker mCherry‐MAP65‐1 was coinfiltrated with M‐YFP into *N. benthamiana* leaves, and at 2 dpi, the infiltrated sections were treated with 20 µM oryzalin and 5 µM Lat B. Controls were treated with an equivalent dilution of DMSO. The confocal microscope images were taken at 3 hr after chemical treatments.

### 4.6 DUAL membrane yeast two‐hybrid assay

The DUAL MYTH system was used following the manufacturer's protocol (Dualsystems Biotech AG). Four AcVD proteins (N, P, P3, and M) were each cloned into the vector pPR3‐N with the N‐terminal half of ubiquitin (NubG), and five proteins (N, P, P3, G, and P6) were separately cloned into the vector pDHB1 with the C‐terminal half of ubiquitin (Cub). The recombined pPR3‐N and pDHB1 plasmids were cotransformed into yeast strain NMY51 (Weidi Bio). The transformed yeast cells were grown on plates containing synthetic dropout medium lacking tryptophan and leucine (SD−LW) (Clontech) at 30 °C for 2–4 days. The surviving clones were transferred to SD−LWHA (Clontech). The plasmid pair pDHB1‐largeT/pDSL‐P53 served as a positive control, and the plasmid pair pDHB1‐largeT/pPR3‐N was used as a negative control.

### 4.7 Virus detection

During 2016–2020, leaf samples were collected from 177 kiwifruit trees, including 51 samples from Hubei Province, 24 samples from Jiangxi Province, 28 samples from Zhejiang Province, 32 samples from Henan Province, 7 samples from Fujian Province, and 36 samples from Chongqing City. Almost all leaf samples exhibited possible virus‐caused symptoms of leaf malformation, chlorotic spots, and/or mosaic symptoms. The presence of AcVD was tested by RT‐PCR using the primer pair 1F/1R (Table S1), which is used to amplify an 847‐bp segment including ORF1, and primer pair 7F/7R (Table S1), which is used to amplify a 544‐bp fragment including ORF7.

## CONFLICT OF INTEREST

The authors have no conflicts of interest.

## Supporting information

**FIGURE S1** Alignment of amino acid sequences of P3 proteins of Actinidia virus D (AcVD) and movement proteins (MP) of two cytorhabdoviruses (LNYV and ADV) and two tobamoviruses (TMV and TVCV) using PROMALS3D. The 30K MP “core” domain along with the secondary consensus structure is denoted by α‐helices (green bars) and β‐elements (red arrows) below the alignment. GenBank accession numbers of sequences used for the analysis are as follows: TMV, tobacco mosaic virus (NC_001367); TVCV, turnip vein‐clearing virus (U03387); ADV, alfalfa dwarf virus (KP205452); LNYV, lettuce necrotic yellows virus (AJ867584)

**FIGURE S2** Amino acid alignment of motifs conserved within RNA‐dependent RNA polymerase (L) encoded by Actinidia virus D (AcVD) and other plant viruses in the family Rhabdoviridae. GenBank accession numbers and abbreviations of viruses used for analysis are shown in Table S2

**FIGURE S3** Subcellular localization assays of Actinidia virus D (AcVD) proteins P, G, N, P6, M, and P3 in epidermal cells of *Nicotiana benthamiana* leaves. The six viral proteins were expressed as fusions to the C‐terminus of eYFP. The fusion proteins H2B‐mCherry and CMV3a‐mCherry were used as nucleus and plasmodesma (PD) markers, respectively. Punctate dots indicating colocalization of AcVD‐P3 with CMV3a‐mCherry at PDs are highlighted by arrowheads. Images were acquired at 2 days after agro‐infiltration under a confocal microscope using a 63×/1.20 water objective. Scale bar = 20 μm

**FIGURE S4** Free eYFP in the cytoplasm and nucleus in *Nicotiana benthamiana* epidermal cells. H2B‐mCherry was used as a nuclear marker. Images were acquired at 2 days after agro‐infiltration under a confocal microscope using a 63×/1.20 water objective. Scale bar = 20 μm

**FIGURE S5** Bimolecular fluorescence complementation (BiFC) assays for Actinidia virus D (AcVD). (a) The nuclear location of the N–M interaction. (b) The interaction signals of N–P, M–P, and M–P6 in an orientation different from that in Figure 5. Glutathione *S*‐transferase (GST) was used as a negative control. The fusion protein H2B‐mCherry was used as a nuclear marker. Images were acquired at 2 days after agro‐infiltration under a confocal microscope using a 63×/1.20 water objective. Scale bar = 20 μm

**TABLE S1** Primers used for the amplification of full‐length cDNA of the Actinidia virus D (AcVD) RNA genome and for the RT‐PCR detection for AcVD in kiwifruit samples

**TABLE S2** The accession numbers of the proteins of plant viruses in the family Rhabdoviridae that were used for phylogenetic analyses

**TABLE S3** Primers designed to amplify ORF1–6 of Actinidia virus D (AcVD) for bimolecular fluorescence complementation assays of the viral proteins

## Data Availability

The data that support the findings of this study are available from the corresponding author upon reasonable request.
