# Supplementary figures and images for: A novel Actinidia cytorhabdovirus characterized using genomic and viral protein interaction features
Source: Mol Plant Pathol. 2021 Jul 20;22(10):1271–87. doi: 10.1111/mpp.13110 (PMC8435229; doi:10.1111/mpp.13110)

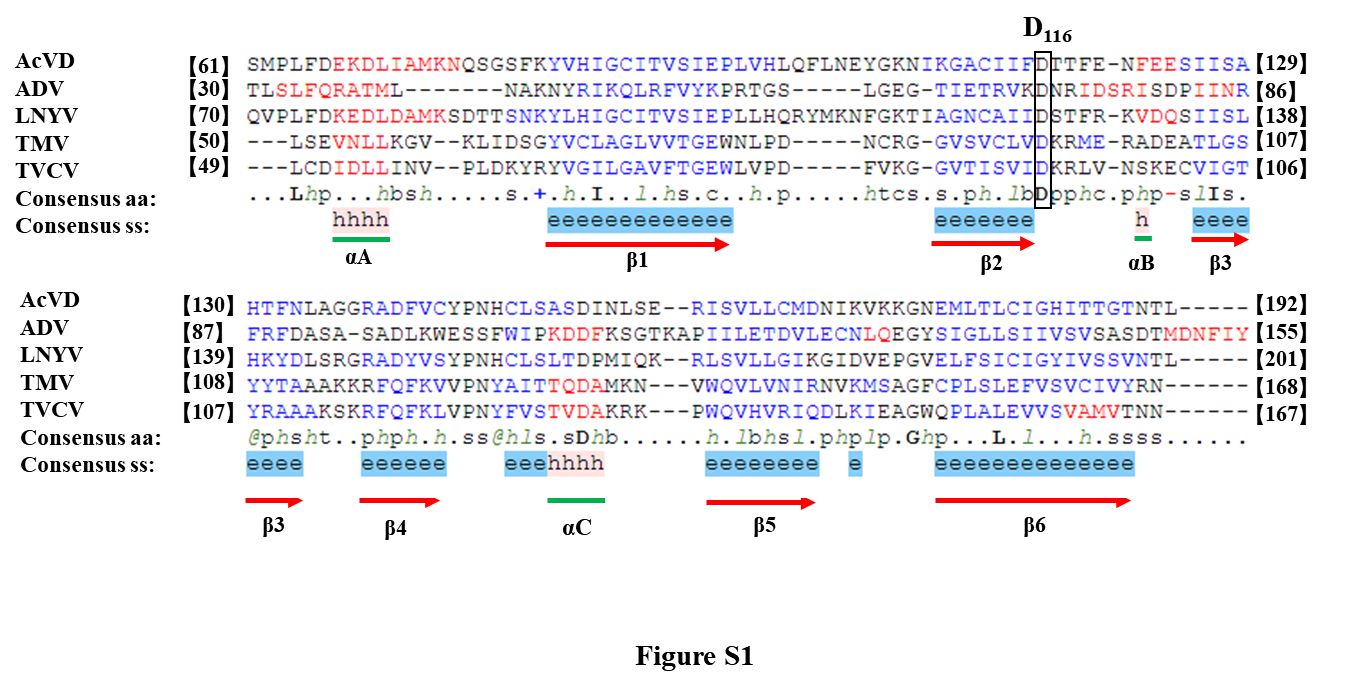

Supplement: Supplementary file 1 — FIGURE S1 Alignment of amino acid sequences of P3 proteins of Actinidia virus D (AcVD) and movement proteins (MP) of two cytorhabdoviruses (LNYV and ADV) and two tobamoviruses (TMV and TVCV) using PROMALS3D. The 30K MP “core” domain along with the secondary consensus structure is denoted by α‐helices (green bars) and β‐elements (red arrows) below the alignment. GenBank accession numbers of sequences used for the analysis are as follows: TMV, tobacco mosaic virus (NC_001367); TVCV, turnip vein‐clearing virus (U03387); ADV, alfalfa dwarf virus (KP205452); LNYV, lettuce necrotic yellows virus (AJ867584) [file MPP-22-1271-s001.docx]

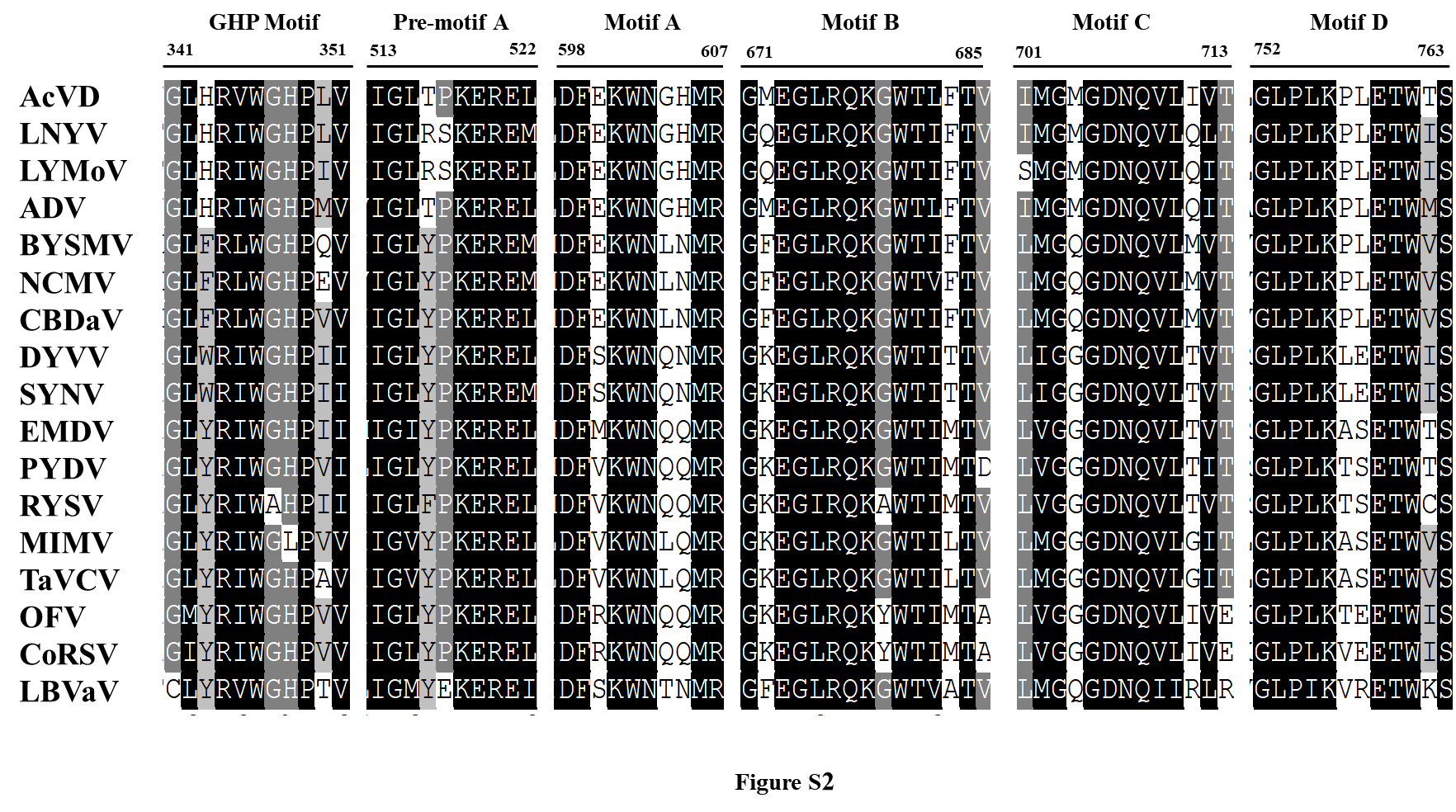

Supplement: Supplementary file 2 — FIGURE S2 Amino acid alignment of motifs conserved within RNA‐dependent RNA polymerase (L) encoded by Actinidia virus D (AcVD) and other plant viruses in the family Rhabdoviridae. GenBank accession numbers and abbreviations of viruses used for analysis are shown in Table S2 [file MPP-22-1271-s002.docx]

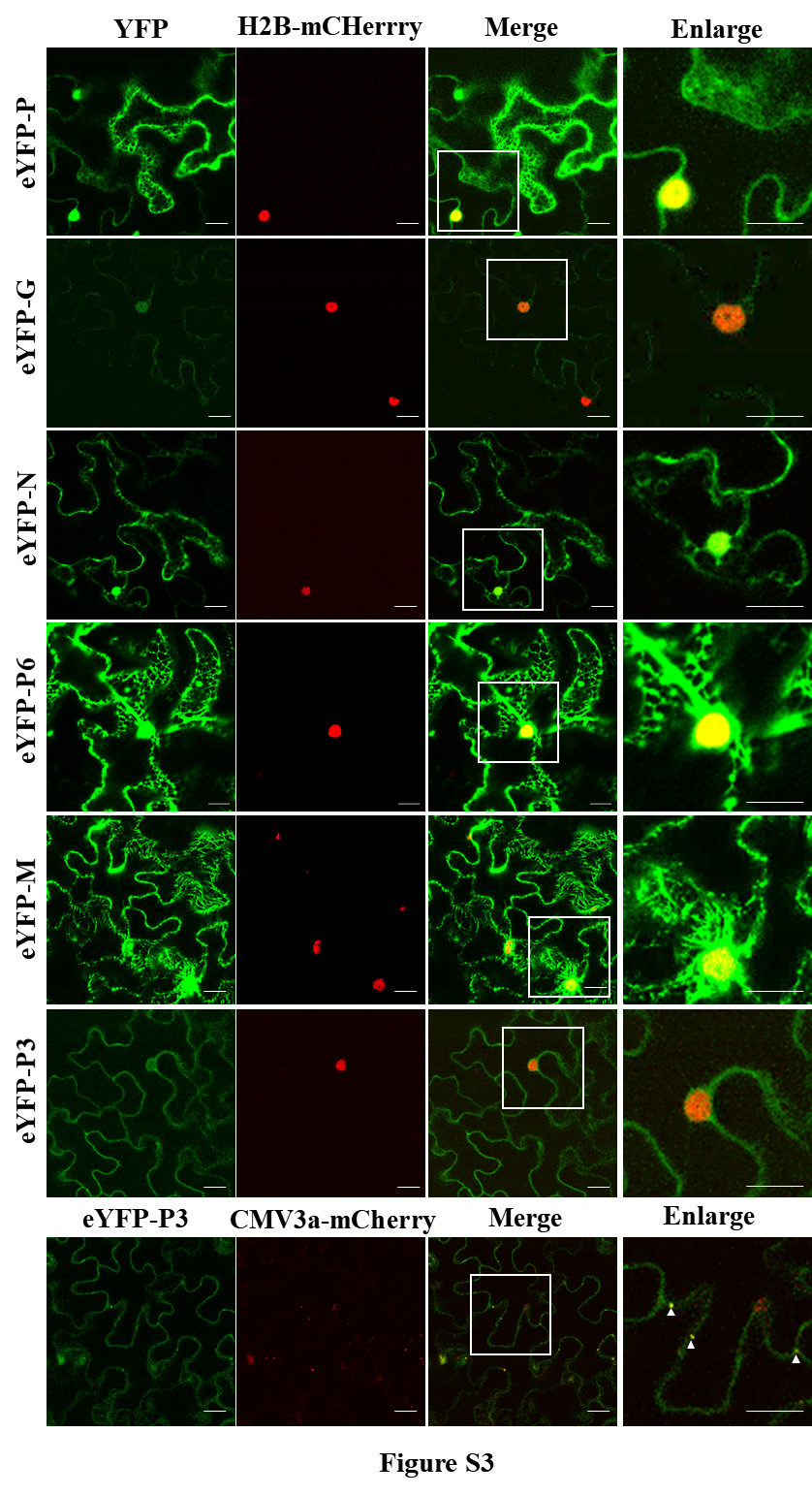

Supplement: Supplementary file 3 — FIGURE S3 Subcellular localization assays of Actinidia virus D (AcVD) proteins P, G, N, P6, M, and P3 in epidermal cells of Nicotiana benthamiana leaves. The six viral proteins were expressed as fusions to the C‐terminus of eYFP. The fusion proteins H2B‐mCherry and CMV3a‐mCherry were used as nucleus and plasmodesma (PD) markers, respectively. Punctate dots indicating colocalization of AcVD‐P3 with CMV3a‐mCherry at PDs are highlighted by arrowheads. Images were acquired at 2 days after agro‐infiltration under a confocal microscope using a 63×/1.20 water objective. Scale bar = 20 μm [file MPP-22-1271-s004.docx]

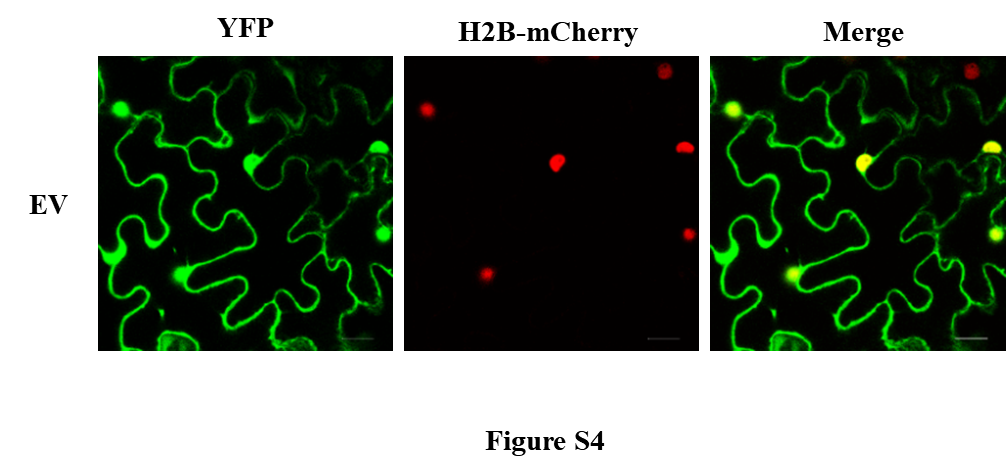

Supplement: Supplementary file 4 — FIGURE S4 Free eYFP in the cytoplasm and nucleus in Nicotiana benthamiana epidermal cells. H2B‐mCherry was used as a nuclear marker. Images were acquired at 2 days after agro‐infiltration under a confocal microscope using a 63×/1.20 water objective. Scale bar = 20 μm [file MPP-22-1271-s008.docx]

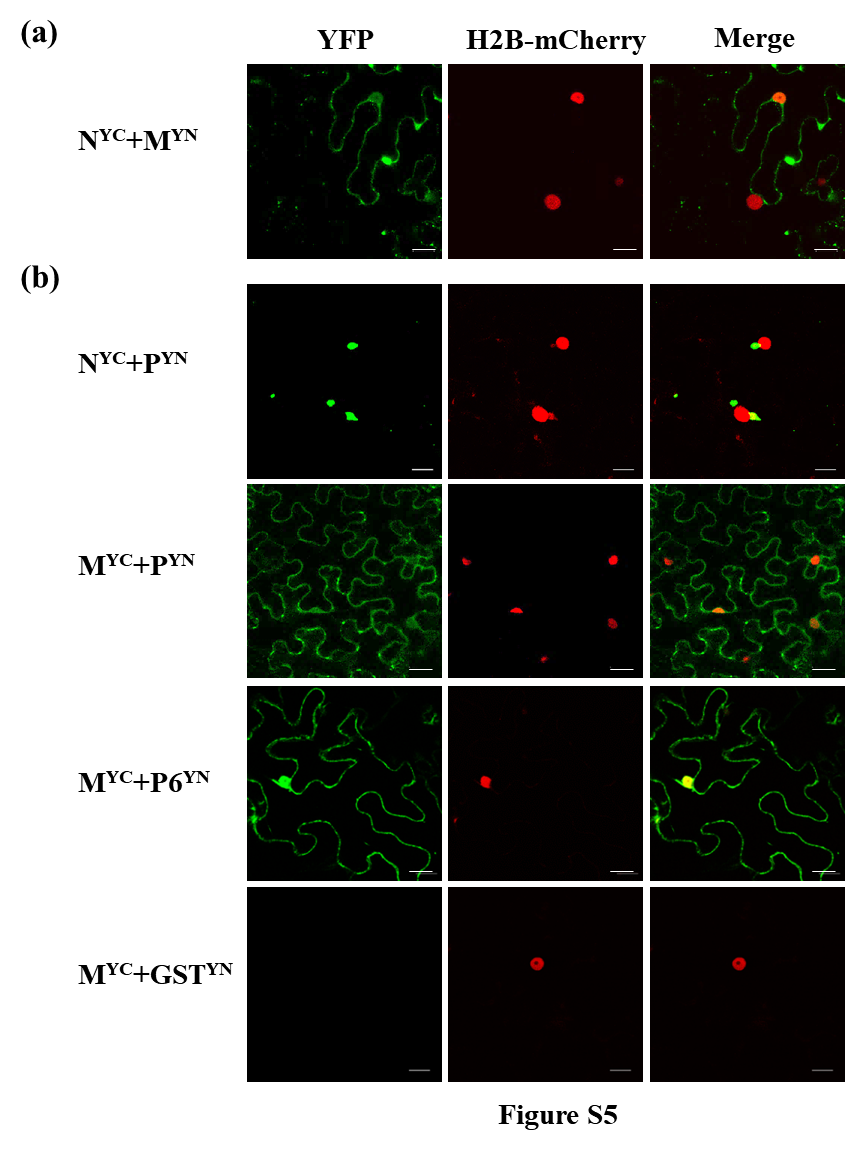

Supplement: Supplementary file 5 — FIGURE S5 Bimolecular fluorescence complementation (BiFC) assays for Actinidia virus D (AcVD). (a) The nuclear location of the N–M interaction. (b) The interaction signals of N–P, M–P, and M–P6 in an orientation different from that in Figure 5. Glutathione S‐transferase (GST) was used as a negative control. The fusion protein H2B‐mCherry was used as a nuclear marker. Images were acquired at 2 days after agro‐infiltration under a confocal microscope using a 63×/1.20 water objective. Scale bar = 20 μm [file MPP-22-1271-s006.docx]
