# Supplementary material for: A novel Actinidia cytorhabdovirus characterized using genomic and viral protein interaction features
Source: Mol Plant Pathol. 2021 Jul 20;22(10):1271–87. doi: 10.1111/mpp.13110 (PMC8435229; doi:10.1111/mpp.13110)
Supplement: Supplementary file 6 — TABLE S1 Primers used for the amplification of full‐length cDNA of the Actinidia virus D (AcVD) RNA genome and for the RT‐PCR detection for AcVD in kiwifruit samples [file MPP-22-1271-s007.docx]

Table S1 Primers used for the amplification of full-length of cDNA Actinidia virus D (AcVD) genome and RT-PCR detection for AcVD in kiwifruit samples

| Primer name | Primer Sequence (5´–3´) | Position | Product size (bp) |
| --- | --- | --- | --- |
| NF | AGGACACTCAAGCCTCAACATC | 233–254 | 1364 |
| NR | CGTTAATCTGTGTACCATATGATACC | 1573–1598 |  |
| 1284F | ACGAAAGTATGAGCTGACAGAG | 1284–1305 | 1677 |
| 2941R | ATAAAGGCATGCTTCGCAAC | 2941–2960 |  |
| 2566F | ATTAGTCGCTTCTTGTGTGCTG | 2704–2725 | 2554 |
| 5350R | TTCTCGAACCTCATGGTCAGC | 5237–5257 |  |
| 5104F | TTTATGCGGCTGACCATGAGG | 5229–5249 | 2503 |
| 7899R | GGGCTTCGTAGGCCTTTATG | 7712–7731 |  |
| 7566F | TGCTACGACAATTCGGCAAC | 7678–7697 | 2814 |
| 10469R | GTAACTGGACTGGGCGTCTC | 10472–10491 |  |
| 10351F | TAACCGTACAATCCGGCTCC | 10426–10445 | 2762 |
| 13220R | AGTATCTCGCCTCTTCCTCAC | 13167–13187 |  |
| 11862F | TCACAAAGCCTACATCTGCCA | 11989–12009 | 1405 |
| 13429R | GGTGTTGATATGGTGGGATGC | 13373–13393 |  |
| 3'-Outer | TACCGTCGTTCCACTAGTGATTT | - | 592 |
| 3'GSP-F1 | TAGAGCACGCCAATGACACG | 542–562 |  |
| 3'-Inner | CGCGGATCCTCCACTAGTGATTTCACTATAGG | - | 470 |
| 3'GSP-F2 | TGAGTTGTTCGACTCCGACT | 438–457 |  |
| 5'-Outer | CATGGCTACATGCTGACAGCCTA | - | 605 |
| 5'GSP-R1 | GCATTCGACACGAAACAAGGT | 12999–13019 |  |
| 5'-Inner | CGCGGATCCACAGCCTACTGATGATCAGTCGATG | - | 570 |
| 'GSP-R2 | ACCTCAAGAGGGAGCAACAT | 13039–13058 |  |
| RT-PCR detection for AcCV | | | |
| 1F | AGGACACTCAAGCCTCAACATC | 233–254 | 847 |
| 1R | TTGTAGGCGACCCACAGAAG | 1060–1079 |  |
| 7F | GAATGTTCTCCCTGATGTC | 8467–8485 | 544 |
| 7R | TGGTTGTCTCCCATCCCCAT | 8991–9010 |  |
