# Supplementary material for: A novel Actinidia cytorhabdovirus characterized using genomic and viral protein interaction features
Source: Mol Plant Pathol. 2021 Jul 20;22(10):1271–87. doi: 10.1111/mpp.13110 (PMC8435229; doi:10.1111/mpp.13110)
Supplement: Supplementary file 7 — TABLE S2 The accession numbers of the proteins of plant viruses in the family Rhabdoviridae that were used for phylogenetic analyses [file MPP-22-1271-s003.docx]

Table S2 The accession number of plant virus in the family *Rhabdoviridae* proteins used for phylogenetic analysis

| Virus name (abbreviation) | Accession number | |
| --- | --- | --- |
|  | L | N |
| Wuhan insect virus 4 (WhIV4) | AJG39179.1 | AJG39174.1 |
| Trifolium pratense virus B (TpVB) | AYH53273.1 | AYH53268.1 |
| lettuce necrotic yellows virus (LNYV) | CAI30426.1 | CAI30421.1 |
| strawberry crinkle cytorhabdovirus (SCV) | AWK49433.1 | AWK49426.1 |
| cabagge cytorhabdovirus 1(CCyV-1) | ATS17313.1 | ATS17308.1 |
| lettuce yellow mottle virus (LYMoV) | ABV56129.1 | ABV56124.1 |
| tomato yellow mottle-associated virus (TYMaV) | YP_009352236.1 | YP_009352242.1 |
| Wuhan insect virus 6 (WhIV6) | AJG39191.1 | AJG39186.1 |
| Trifolium pratense virus A (TpVA) | AYH53279.1 | AYH53274.1 |
| alfalfa dwarf virus (ADV) | AKD44217.1 | AKD44211.1 |
| raspberry vein chlorosis virus (RVCV) | QBS46644.1 | QBS46637.1 |
| persimmon virus A(PeVA) | YP_006576506.2 | YP_006576501.2 |
| Wuhan insect virus 5 (WhIV5) | AJG39185.1 | AJG39180.1 |
| barley yellow striate mosaic virus (BYSMV) | AJP67524.1 | AJP67515.1 |
| maize yellow striate virus (MYSV) | ATN96443.1 | ATN96434.1 |
| northern cereal mosaic virus (NCMV) | BAA95352.1 | BAA95345.1 |
| maize-associated cytorhabdovirus (MaCyV) | ARS22495.1 | ARS22490.1 |
| rice stripe mosaic virus (RSMV) | AZB50452.1 | AZB50446.1 |
| colocasia bobone disease-associated virus (CBDaV) | ALU34427.1 | ALU34429.1 |
| yerba mate chlorosis-associated virus (YmCaV) | ARA91091.1 | AYR67253.1 |
| Papaya virus E (PpVE) | AYD37624.1 | AYD37618.1 |
| Trichosanthes associated rhabdovirus 1 (TrAV-1) | DAC81998.1 | DAC81993.1 |
| paper mulberry mosaic-associated virus (PMuMaV) | QNO38995.1 | QNO38990.1 |
| cucurbit cytorhabdovirus 1 (CuCV-1) | QLT57530.1 | QLT57524.1 |
| strawberry cytorhabdovirus 1(StrV-1) | QGN65754.1 | QGN65746.1 |
| potato yellow dwarf virus (PYDV) | ADE45274.1 | ADE45268.1 |
| eggplant mottled dwarf virus (EMDV) | AHN10100.1 | AHN1094.1 |
| Maize mosaic virus (MMV) | AAT66757.1 | AAT66752.1 |
| rice yellow stunt virus (RYSV) | BAA25160.1 | BAA25154.1 |
| datura yellow vein virus (DYVV) | AKH61406.1 | AKH61401.1 |
| maize fine streak virus (MFSV) | AAT66751.1 | AAT66750.1 |
| sonchus yellow net virus (SYNV, ) | AAA50385.1 | AAA50380.1 |
| coffee ringspot virus (CoRSV) | AHH44830.1 | AHH44825.1 |
| lettuce big-vein associated virus (LBVaV) | BAC16226.1 | BAC16230.1 |
