# Supplementary material for: A novel Actinidia cytorhabdovirus characterized using genomic and viral protein interaction features
Source: Mol Plant Pathol. 2021 Jul 20;22(10):1271–87. doi: 10.1111/mpp.13110 (PMC8435229; doi:10.1111/mpp.13110)
Supplement: Supplementary file 8 — TABLE S3 Primers designed to amplify ORF1–6 of Actinidia virus D (AcVD) for bimolecular fluorescence complementation assays of the viral proteins [file MPP-22-1271-s005.docx]

Table S3 Primers designed for amplification of the ORF1-6 of Actinidia virus D (AcVD) isolate JS27.

| ORF | Primer | Sequence ^a^ (5´–3´) | Position | Product size |
| --- | --- | --- | --- | --- |
|  |  |  | (nt) | (bp) |
| ORF1 | P1-F | AAAAAGCAGGCTCCATGGCAGAATTCACAACAG | 160–178 | 1434 |
|  | P1-R | AGAAAGCTGGGTCATCTGTGTACCATATGATAC | 1574–1593 |  |
| ORF2 | P2-F | AAAAAGCAGGCTCCATGGAAAGTGAATCCGAC | 1732–1749 | 912 |
|  | P2-R | AGAAAGCTGGGTC AGAAAATAACTCCTCCTCAAG | 2623–2643 |  |
| ORF3 | P3-F | AAAAAGCAGGCTCC ATGGATTCAAATGGTATC | 2768–2785 | 1092 |
|  | P3-R | AGAAAGCTGGGTCCATTAGTTTTGAATACTCC | 3841–3858 |  |
| ORF4 | P4-F | AAAAAGCAGGCTCCATGGCTGATAACATGAGTTC | 1317–1392 | 570 |
|  | P4-R | AGAAAGCTGGGTCCAGGCTTTATAACATCAGTGC | 4531–4450 |  |
| ORF5 | P5-F | AAAAAGCAGGCTCCATGAATCGCGCCATGGTATG | 1131–1151 | 1656 |
|  | P5-R | AGAAAGCTGGGTCAATGAATTCCATCTGATAT | 318–336 |  |
| ORF6 | P6-F | AAAAAGCAGGCTCCATGTTCTTAGAGAGAGGAG | 6560–6578 | 180 |
|  | P6-R | AGAAAGCTGGGTCCAAGTAATGGATATAGACG | 6721–6739 |  |

a: The artificially added nucleotides used for recombination reaction were underlined.
